# Supplementary material for: Parasitic plasmids are anchored to inactive regions of eukaryotic chromosomes through a nucleosome signal
Source: EMBO J. 2025 Feb 27;44(7):2134–56. doi: 10.1038/s44318-025-00389-1 (PMC11962162; doi:10.1038/s44318-025-00389-1)
Supplement: Supplementary file 1 — Appendix [file 44318_2025_389_MOESM1_ESM.pdf]

Appendix for

Parasitic plasmids are anchored to inactive regions of eukaryotic chromosomes through a nucleosome signal

Fabien Girard[1,2,3], Antoine Even[4], Agnès Thierry[1], Myriam Ruault[4], Léa Meneu[1,2], Pauline Larrous[1,2], Mickaël Garnier[4], Sandrine Adiba[5], Angela Taddei[4], Romain Koszul[1,#], Axel Cournac[1,#]

1 Institut Pasteur, CNRS UMR 3525, Université Paris Cité, Unité Régulation Spatiale des Génomes, 75015 Paris, France

2 Sorbonne Université, Collège Doctoral, F-75005, Paris, France

3 Department of Biology, École Normale Supérieure Paris-Saclay Université Paris-Saclay, Gif-sur-Yvette 91190, France.

4 Institut Curie, Université PSL, Sorbonne University, CNRS, Nuclear Dynamics, Paris, France.

5 Institut de Biologie de l'Ecole Normale Supérieure, Département de Biologie, CNRS, Ecole Normale Supérieure, CNRS, INSERM, PSL Research University, Paris, France

# corresponding authors: romain.koszul@pasteur.fr, axel.cournac@pasteur.fr

Table of contents

Appendix Figure S1: Genomic signals along the 2µ plasmid of Saccharomyces cerevisiae. page 3

Appendix Figure S2: ChIP-seq of Rep1 protein from the 2µ plasmid. page 4

Appendix Figure S3: Contact signal of the control plasmids along the 16 chromosomes of *S. cerevisiae*. page 5

Appendix Figure S4: Percentage of reads coming from 2µ plasmid sequence in WT and various mutants computed from Hi-C libraries. page 6

Appendix Figure S5: Contact signal of 2µ plasmid mutants. page 7

Appendix Figure S6: Contact of the 2µ plasmid during mitotic and meiotic cell cycles. page 8

Appendix Figure S7: Statistical analyses on the size and transcription level of genes contacted by 2µ plasmid. page 9

Appendix Figure S8: Contact behavior for the identified loci contacted by 2µ plasmid. page 11

Appendix Figure S9: Contact signal of the 2µ plasmid with exogenous sequences. page 12

Appendix Figure S10: Microscopy FISH analysis showing the colocalisation of 2µ plasmid with Mmyco supplementary chromosome. page 13

Appendix Figure S11: Averaged plot around contacted regions by 2µ for various genomic signals. page 14

Appendix Figure S12: Contact signal of 2µ plasmid in epigenetic marks and chromatin remodelers mutants. page 15

Appendix Table S1: List of strains used in the present study. page 17

Appendix Table S2: List of plasmids used in this study. page 19

Appendix Table S3: Genomic datasets generated in this study. page 20

Appendix Table S4: Contact datasets analysed in the present study. page 22

Appendix Table S5: Genomic datasets (other than contact data) analyzed in the present study. page 24

Appendix Table S6: Set of the 73 hotspots of contact of 2µ plasmid automatically detected in WT, log phase condition. page 25

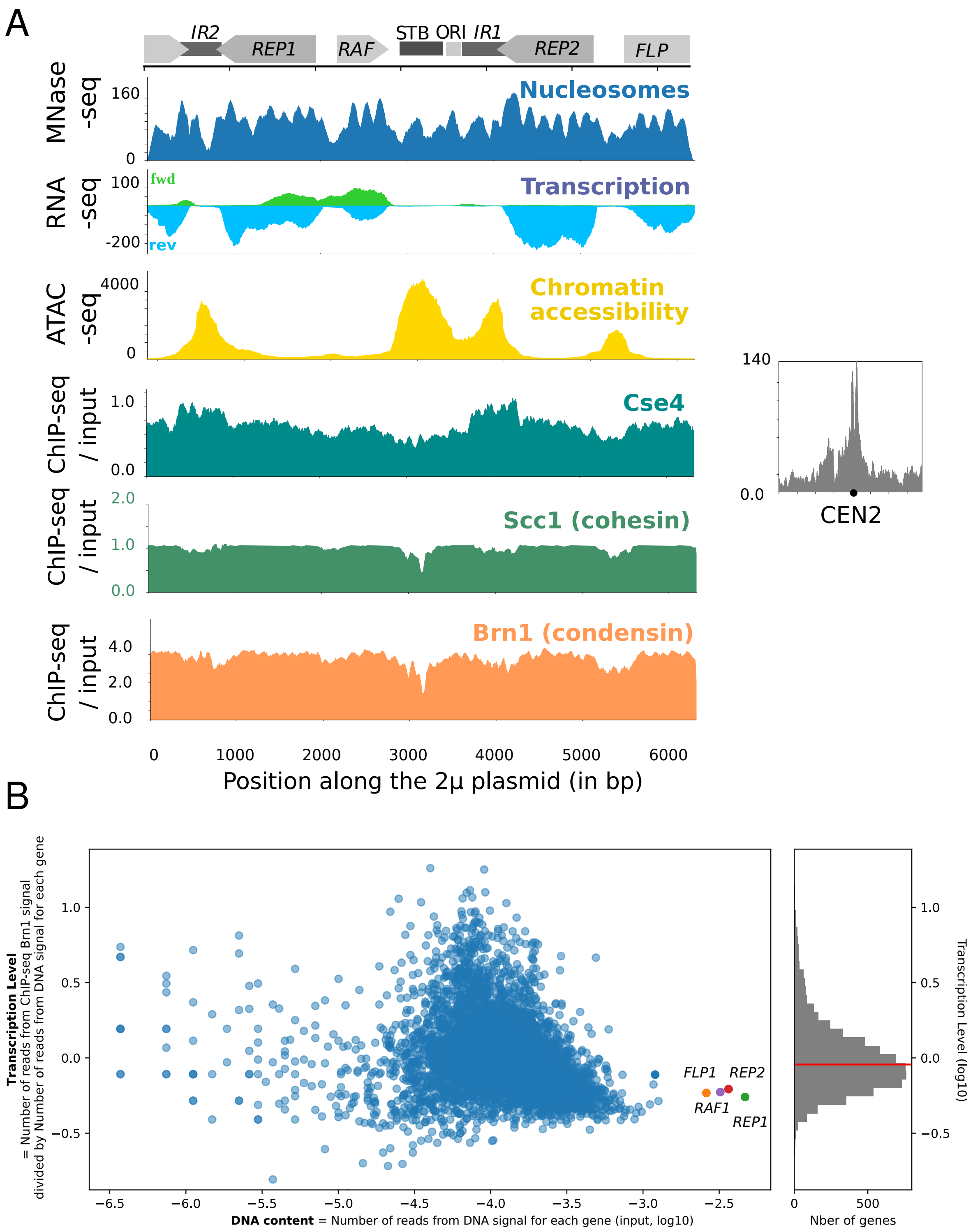

Appendix Figure S1: Genomic signals along the 2*μ* plasmid of *Saccharomyces cerevisiae.*

A, Nucleosomes signal along the 2*μ* plasmid from H3 chemical cleavage data in counts per million (CPM) (Chereji *et al*, 2018). Transcription signal along the 2*μ* plasmid from RNA-seq data of (Garcia-Luis *et al*, 2019) in counts per million (CPM). Chromatin accessibility signal along the 2*μ* plasmid from ATAC-seq data of (Sánchez-Gaya *et al*, 2018) in counts per million (CPM), same as Figure 1. Protein occupancy of Cse4 along the 2*μ* plasmid, from ChIP-seq data of (Au *et al*, 2020) as well at the position of centromere of chromosome II (y-axis not as the same scale). Scc1 protein occupancy (part of cohesin complex (Verzijlbergen *et al*, 2014)) and Brn1 (subunit of condensin (Swygert *et al*, 2019)). **B,** Transcription level for each gene of the 2μ plasmid and the genes of *S. cerevisiae* in function of their DNA content. On the right, the distribution of transcription levels is shown for all genes with median (red line).

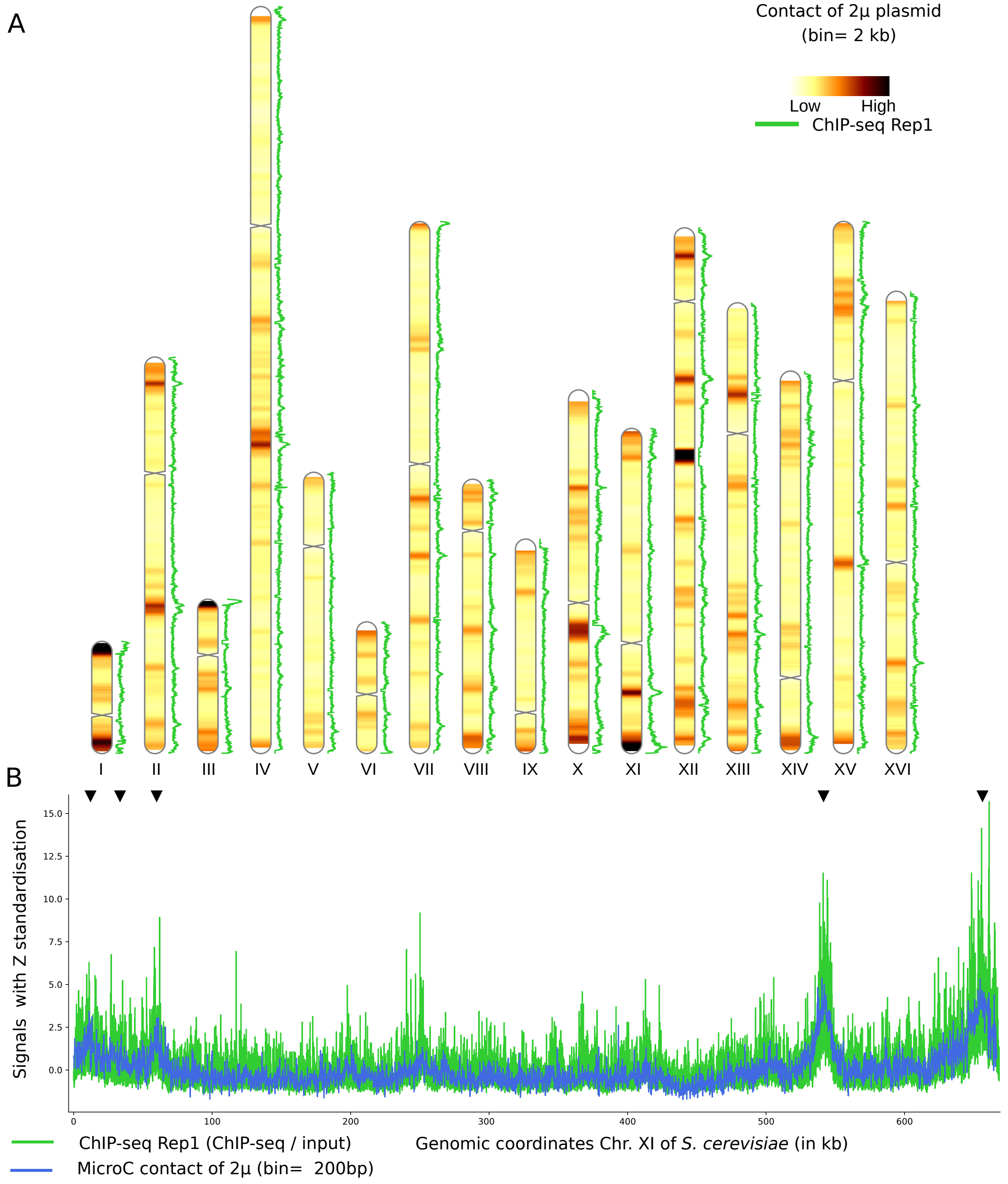

Appendix Figure S2: ChIP-seq of Rep1 protein from the 2*µ* plasmid.

A, ChIP-seq of Rep1 protein along with the contact profile of 2*µ* plasmid with the chromosomes of *S. cerevisiae* (chromosomal heatmap diagram). **B**, ChIP-seq of Rep1 protein (IP/input) and contact profile of 2*µ* plasmid binned at 200 bp, (MicroC data from (Swygert *et al*, 2019)) for the chromosome XI of *S. cerevisiae*. Both signals were Z standardized i.e $z = (x-\mu)/\sigma$, where x is the ChIP-seq signal value (ChIP/input) or contact score, μ is the mean and σ is the standard deviation of each signal. Black triangle indicates detected peaks in contact signal of 2µ plasmid.

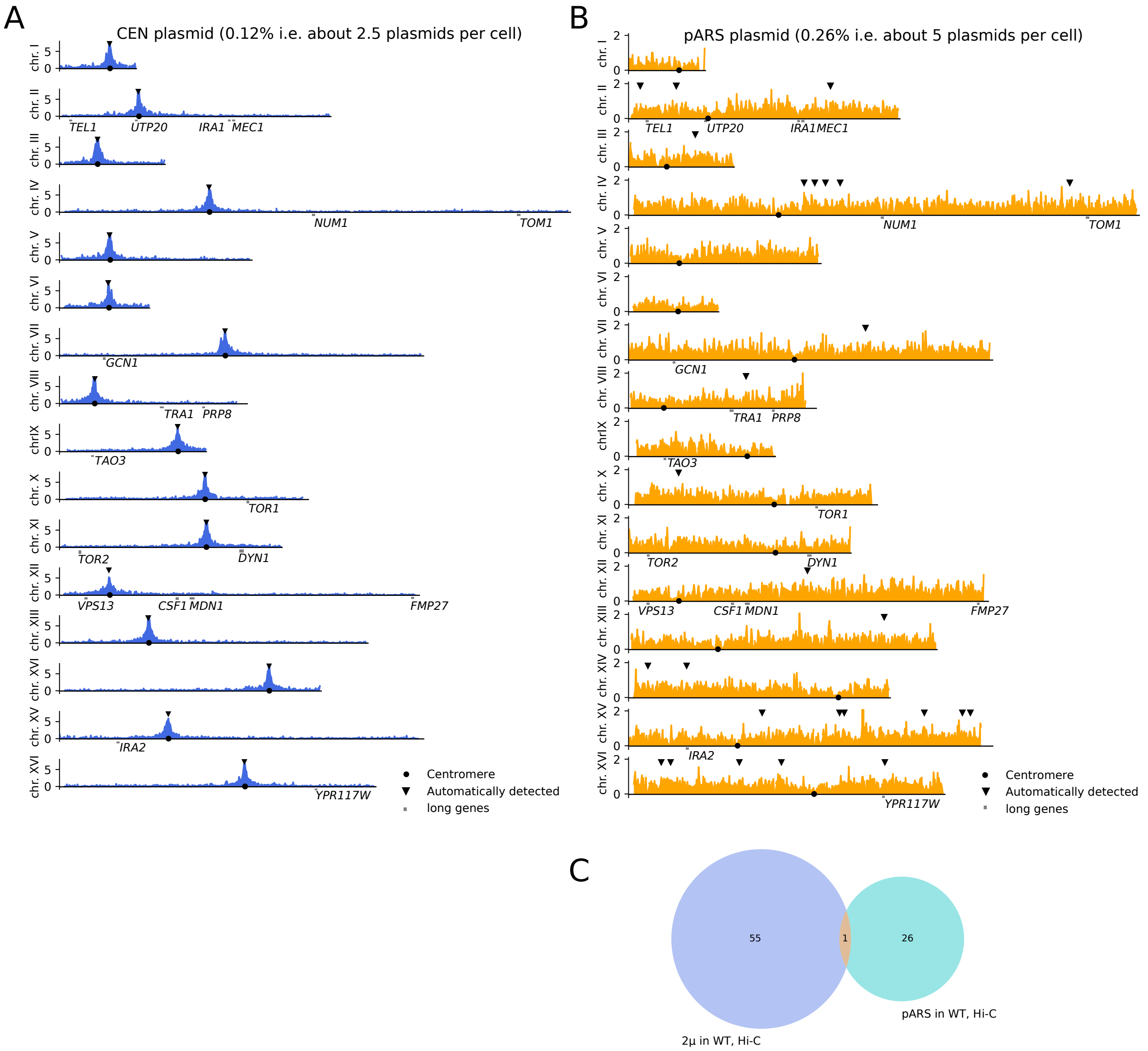

Appendix Figure S3: Contact signal of the control plasmids along the 16 chromosomes of *S. cerevisiae*.

A, Contact signal of the yeast centromeric Plasmid (YCp) pRS416 along the 16 chromosomes of *S. cerevisiae*. The Hi-C contact signal is binned at 2 kb, and names of genes with size >7 kb are annotated. Automatically detected peaks of contact were annotated with black triangles. **B,** Contact signal of a replicative plasmid devoid of centromere (pARS) and 2μ system along the 16 chromosomes of *S. cerevisiae*. The contact signal is binned at 2 kb, names of genes with size >7 kb are annotated. **C,** Venn diagram of the detected peaks of 2μ plasmid in WT background and detected peaks of pARS plasmid in WT background, Hi-C (asynchronous cells).

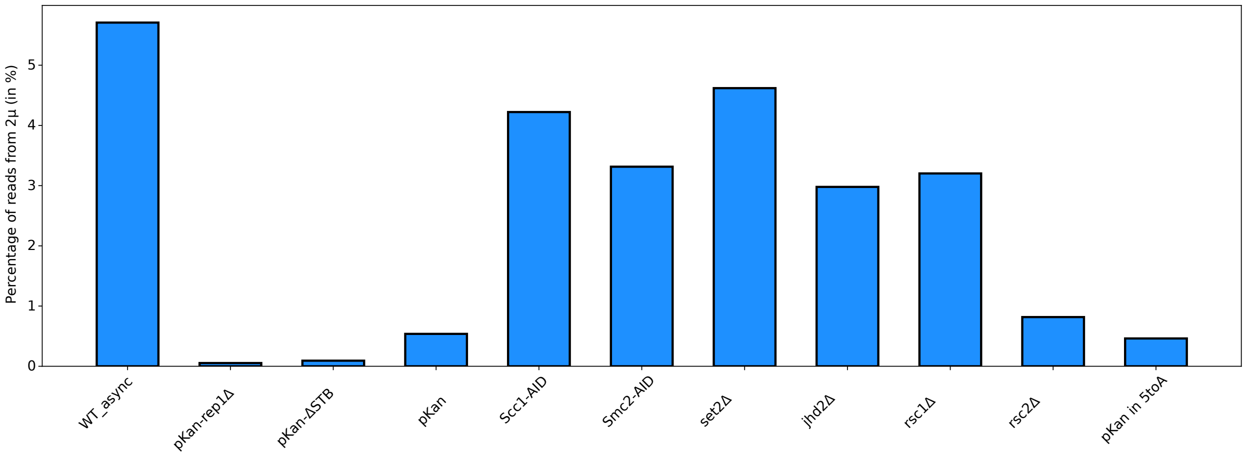

Appendix Figure S4: Percentage of reads coming from 2µ plasmid sequence in WT and various mutants computed from Hi-C libraries.

For mutants of *S. cerevisiae* with Scc1 degron mutant (sub-unit of cohesin, AID system) data of (Dauban *et al*, 2020), were used and for Smc2 degron mutant (sub-unit of condensin, AID system) data from (Guérin *et al*, 2019)**,** all other data were generated from this study.

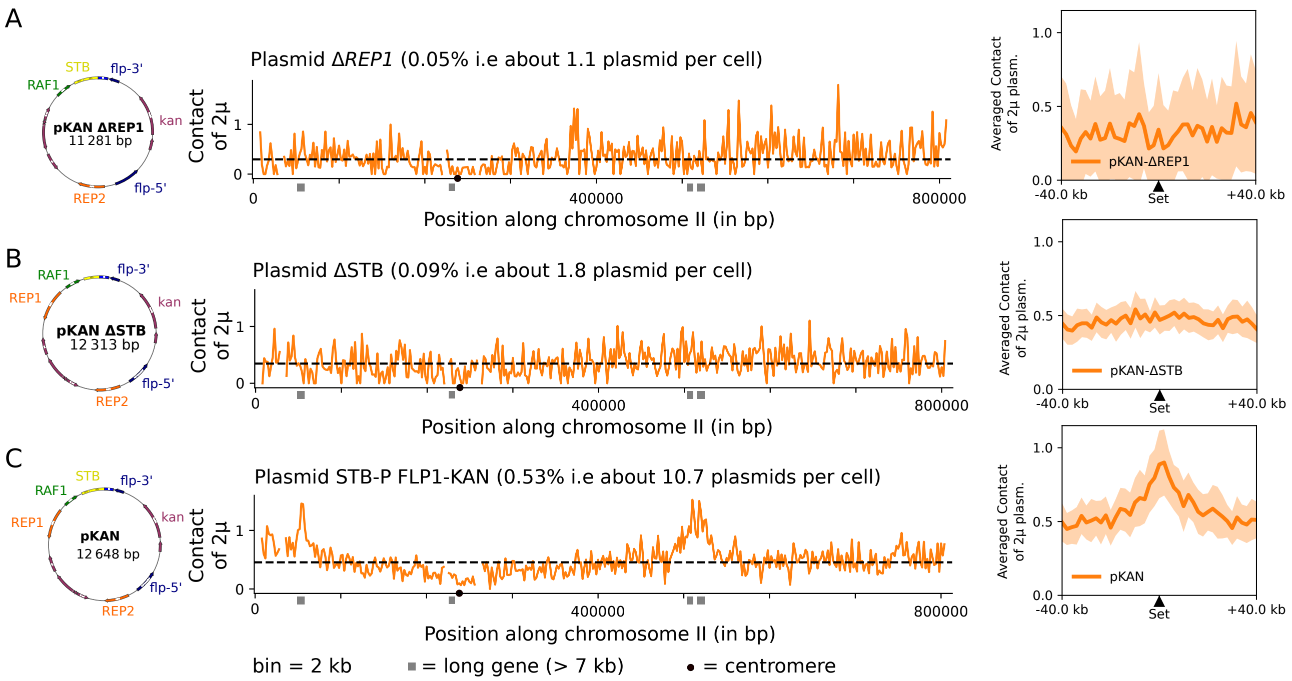

Appendix Figure S5: Contact signal of 2μ plasmid mutants.

A, Contact signal of the Δ*REP1* mutant 2μ plasmid along the chromosome II of *S. cerevisiae* and the averaged contact signal on the hot spots of contact detected in WT, log phase condition. **B**, Same for the ΔSTB mutant 2μ plasmid. **C**, Same for the STB-P 2μ mutant plasmid.

Plasmids pKan-Δ*REP1* and pKan-ΔSTB are derived from the initial plasmid pKan whose STB region contains only the STB-P part. STB-P stands for *STB-proximal*, so named for its positioning relative to the single origin of replication (*ORI)* on the 2µ plasmid (McQuaid *et al*, 2019).

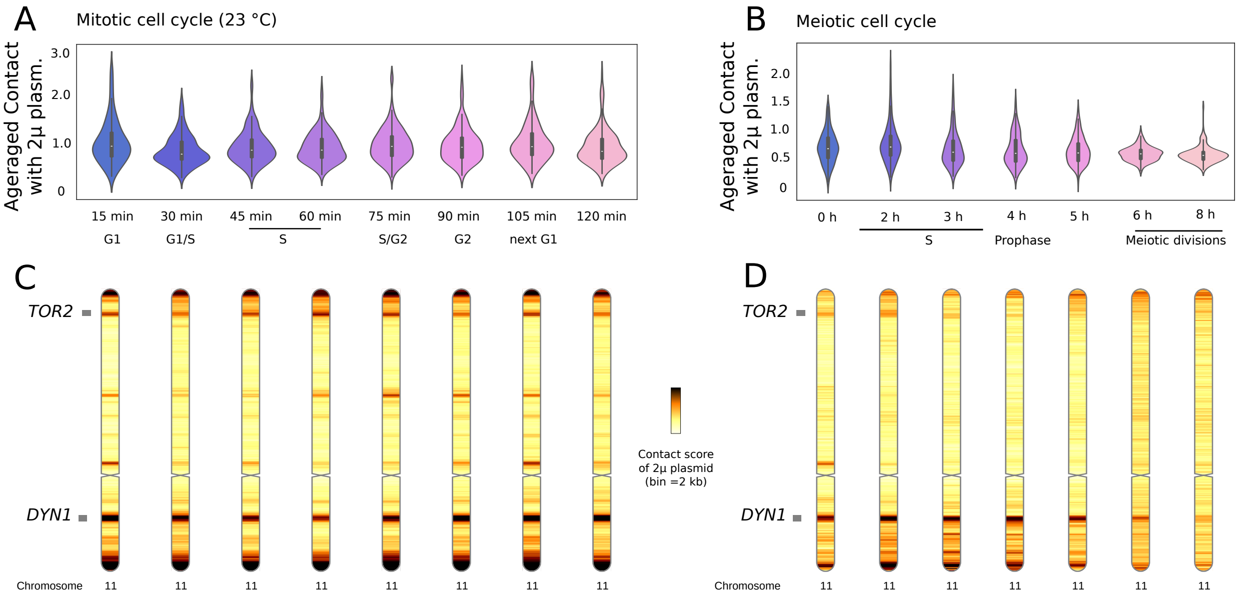

Appendix Figure S6: Contact of the 2μ plasmid during mitotic and meiotic cell cycles.

A, Distribution of contact values of identified hotspots contacted by the 2μ plasmid identified in WT, log phase during the mitotic cell cycle (contact data reanalysed from (Costantino *et al*, 2020)). **B**, Distribution of contact values of the identified hotspots contacted by the 2μ plasmid during the meiotic cell cycle (contact data reanalysed from (Schalbetter *et al*, 2019)). **C**, Example of contact profile of 2μ plasmid with chromosome XI during mitotic cell cycle. **D**, Example of contact profile of 2μ plasmid with chromosome XI during meiotic cell cycle. Long genes (*TOR2*, *DYN1* with size > 7 kb) are annotated.

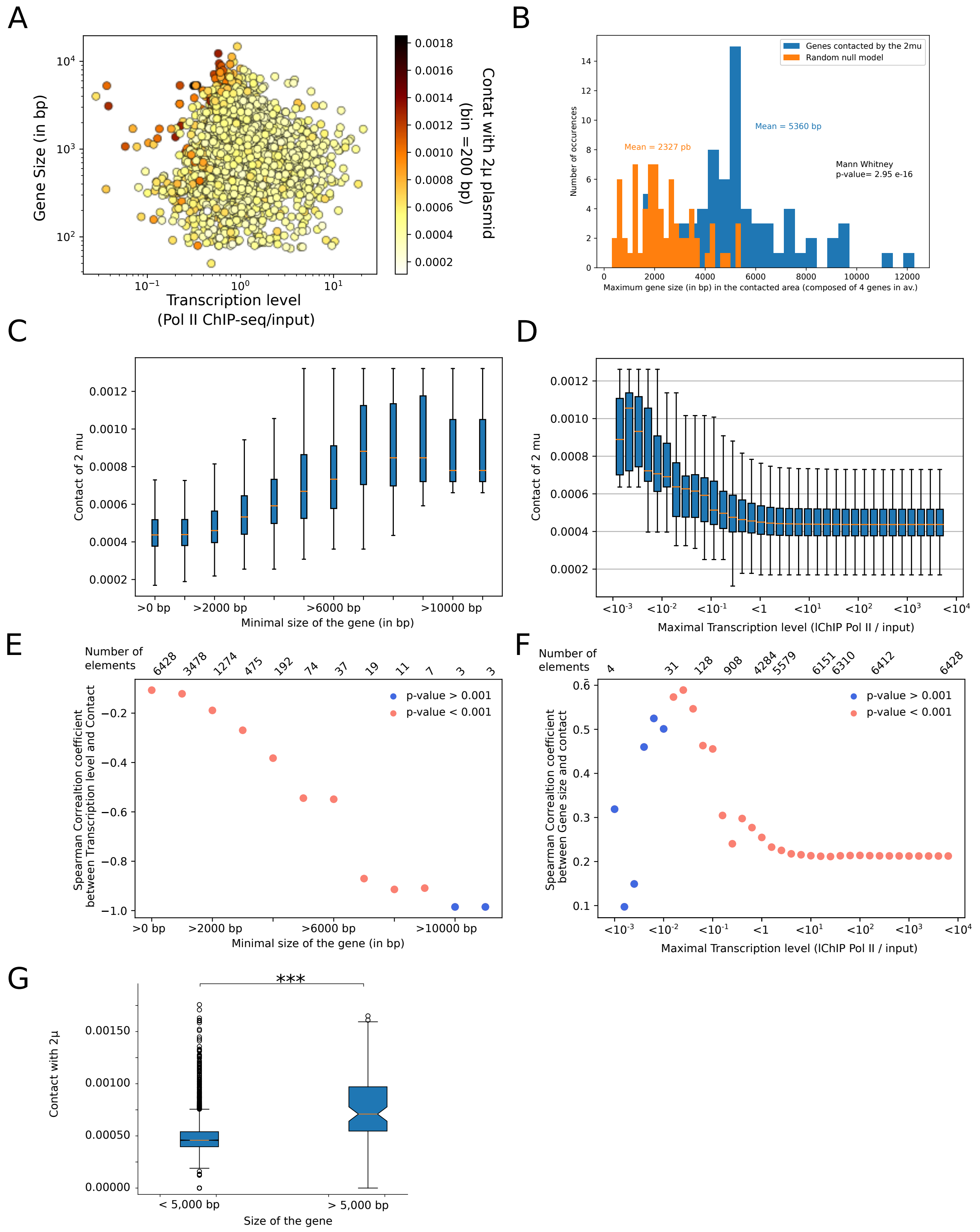

Appendix Figure S7: Statistical analyses on the size and transcription level of genes contacted by 2μ plasmid.

A, Scatter plot for all genes of *S. cerevisiae* represented in function of their transcription level (x-axis), their size in bp (y-axis) and their level of contact with 2μ plasmid represented by their color (colorbar on the left). MicroC data were reanalysed from (Swygert *et al*, 2019). **B**, Distribution of maximum sizes of genes from loci contacted by 2μ plasmid and from a random group of loci with the associated statistical test. **C**, Contact

level of 2μ plasmid in function of the minimal size of gene (in bp). **D**, Contact level of 2μ plasmid in function of the maximal transcription level (ChIP-seq data of Rpb3, sub-unit of PolII, (Swygert *et al*, 2019)). **E**, Spearman correlation coefficient between transcription level and contact with 2μ plasmid in function of the minimal size of gene (in bp). **F**, Spearman correlation coefficient between gene size and contact with 2μ plasmid in function of the maximal transcription level (ChIP-seq data of Rpb3, subunit of PolII from (Swygert *et al*, 2019)). The number of elements to compute the Pearson correlation is given at the top of the plot.**G**, Box plot of the contact scores for 2 groups of genes with size > or < 5,000 bp. Mann Whitney test was applied (p-value<0.001).

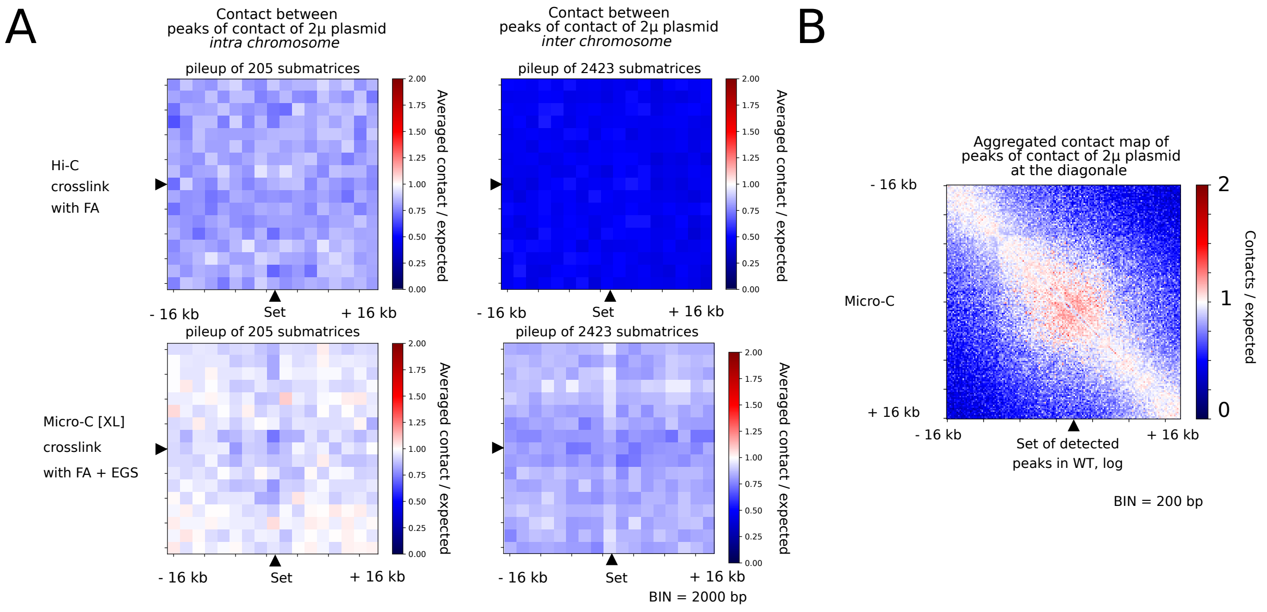

Appendix Figure S8: Contact behavior for the identified loci contacted by 2*μ* plasmid.A, Agglomerated plot between pairs of loci contacted by 2*μ* plasmid belonging to the same chromosome (left) or belonging to different chromosomes (right) for two different contact technologies: Hi-C (top) and MicroC with dual crosslink (bottom) (Swygert *et al*, 2019). The signal represents the ratio between the contact measured between loci contacted by 2*μ* plasmid over random pairs separated by same genomic distances (Matthey-Doret *et al*, 2020). **B**, Agglomerated plot at the diagonal for the identified loci contacted by 2*μ* plasmid with bins of 200 bp (MicroC data reanalysed from (Swygert *et al*, 2019)).

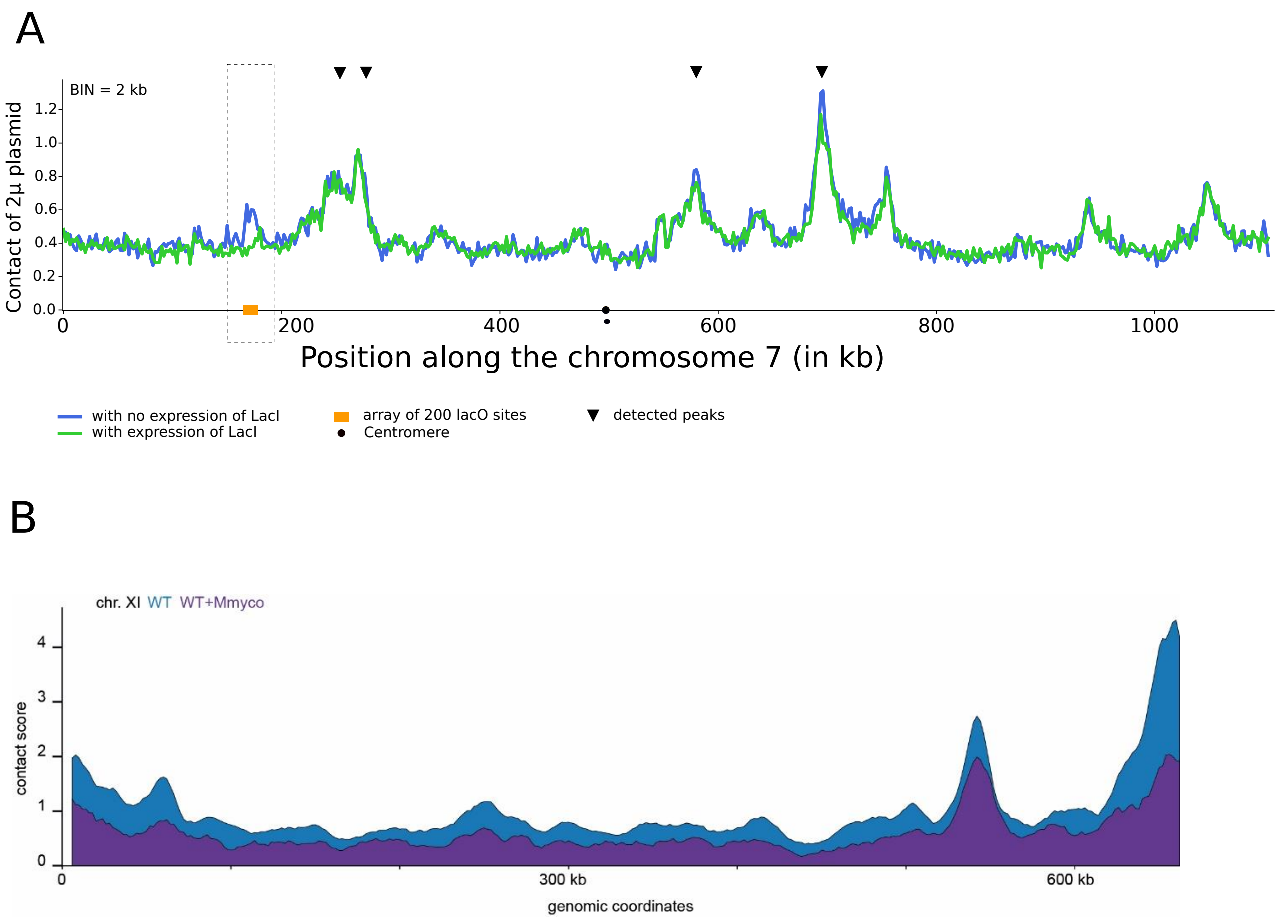

Appendix Figure S9: Contact signal of the 2µ plasmid with exogenous sequences.

A, Contact signal of the 2µ plasmid with an array of 200 LacO binding sites without and with expression of LacI. The array of 200 LacO binding sites is represented by an orange box. Automatically detected peaks are represented by black triangles. Contact enrichment on the LacO binding site array is visible only in the condition without LacI expression.

B, The contact signal of the 2µ plasmid on the host chromosome XI is plotted in WT condition (blue) and in a strain where Mmyco supernumerary artificial chromosome is present (purple). The overall signal along chromosome XI is diminished in the presence of the artificial chromosome.

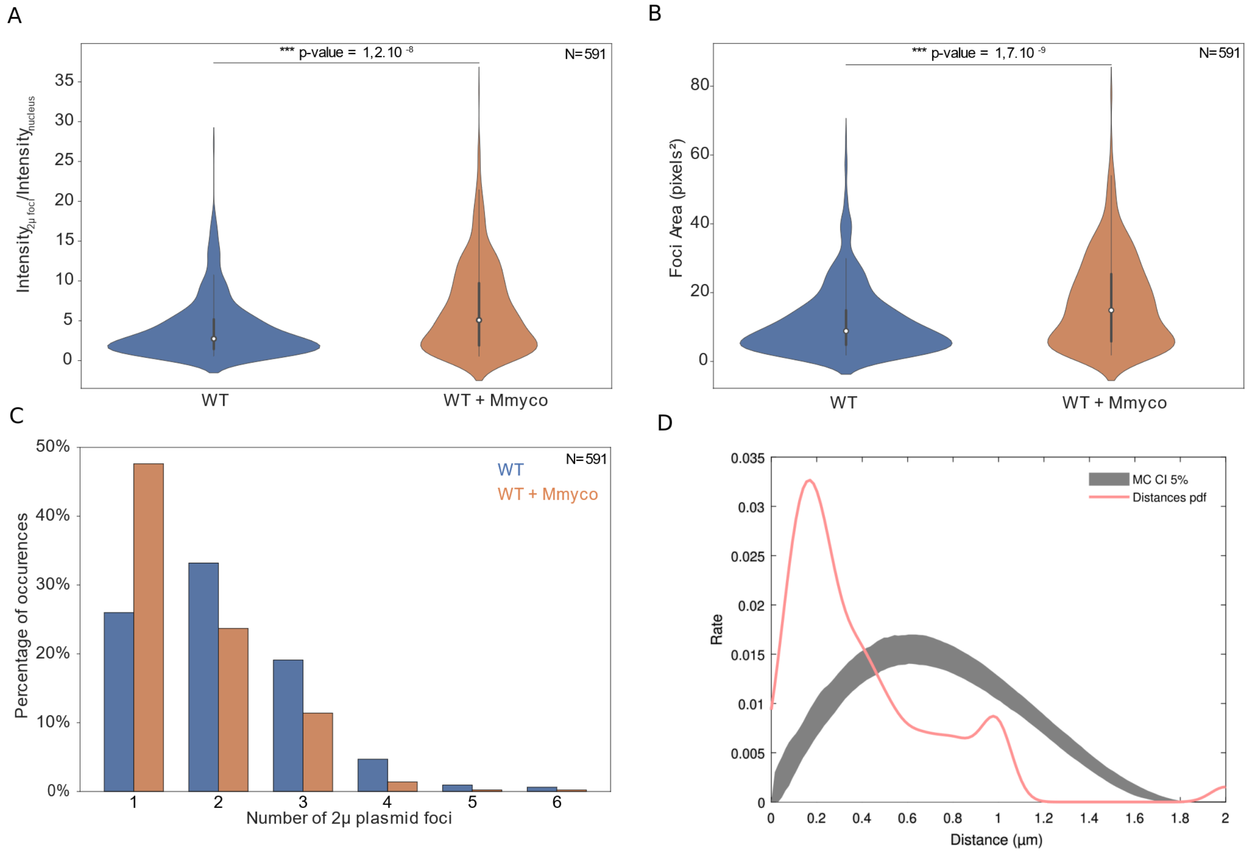

Appendix Figure S10: Microscopy FISH analysis showing the colocalisation of 2µ plasmid with Mmyco supplementary chromosome.

Quantification of FISH images for WT cells (blue) and WT with Mmyco supplementary chromosome cells (orange) of the intensity of the fluorescence from the 2µ plasmid.

A, Intensity of 2µ focus normalized by the intensity of the 2µ signal from the whole nucleus. **B,** Area of the 2µ plasmid foci. **C,** Distribution of the number of 2µ plasmid foci per cell. **D,** Distribution of distances in µm between the center of mass of the Mmyco supplementary chromosome and the center of mass of the 2µ plasmid (red line) and comparison with a null model consisting in a random distribution of foci positions in the nucleus (grey line, MC-CI 5% : Monte Carlo approach with confidence interval at 5%).

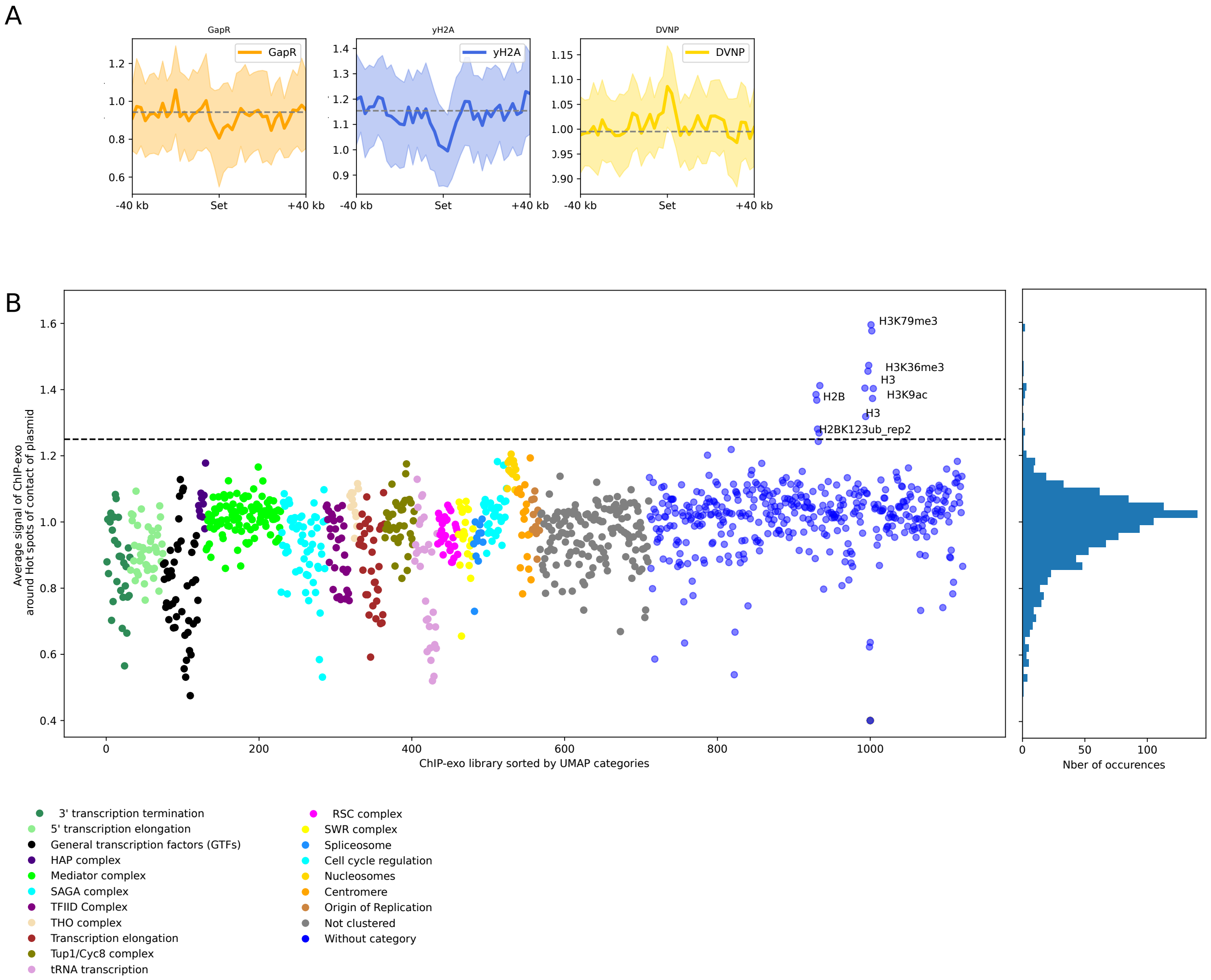

Appendix Figure S11: Averaged plot around contacted regions by 2µ for various genomic signals.

A, GapR ChIP-seq signal giving the presence of positive supercoiling is depleted around regions contacted by the 2µ. γH2A ChIP-seq signal giving the presence of the histone mark γH2A which is associated with yeast heterochromatin is depleted around regions contacted by the 2µ. Dinoflagellate-viral-nucleoproteins (DVNPs) expressed in yeast show enrichment in regions contacted by 2µ.

B, Average value of ChIP-exo signal at the hotspots of contact for 1251 ChIP-exo libraries (Rossi *et al*, 2021) sorted by general categories with alternative category annotation. Right, distribution of scores for the libraries. The dotted line defines the threshold (1.25) above which the p-value of the observed enrichment is < 0.01.

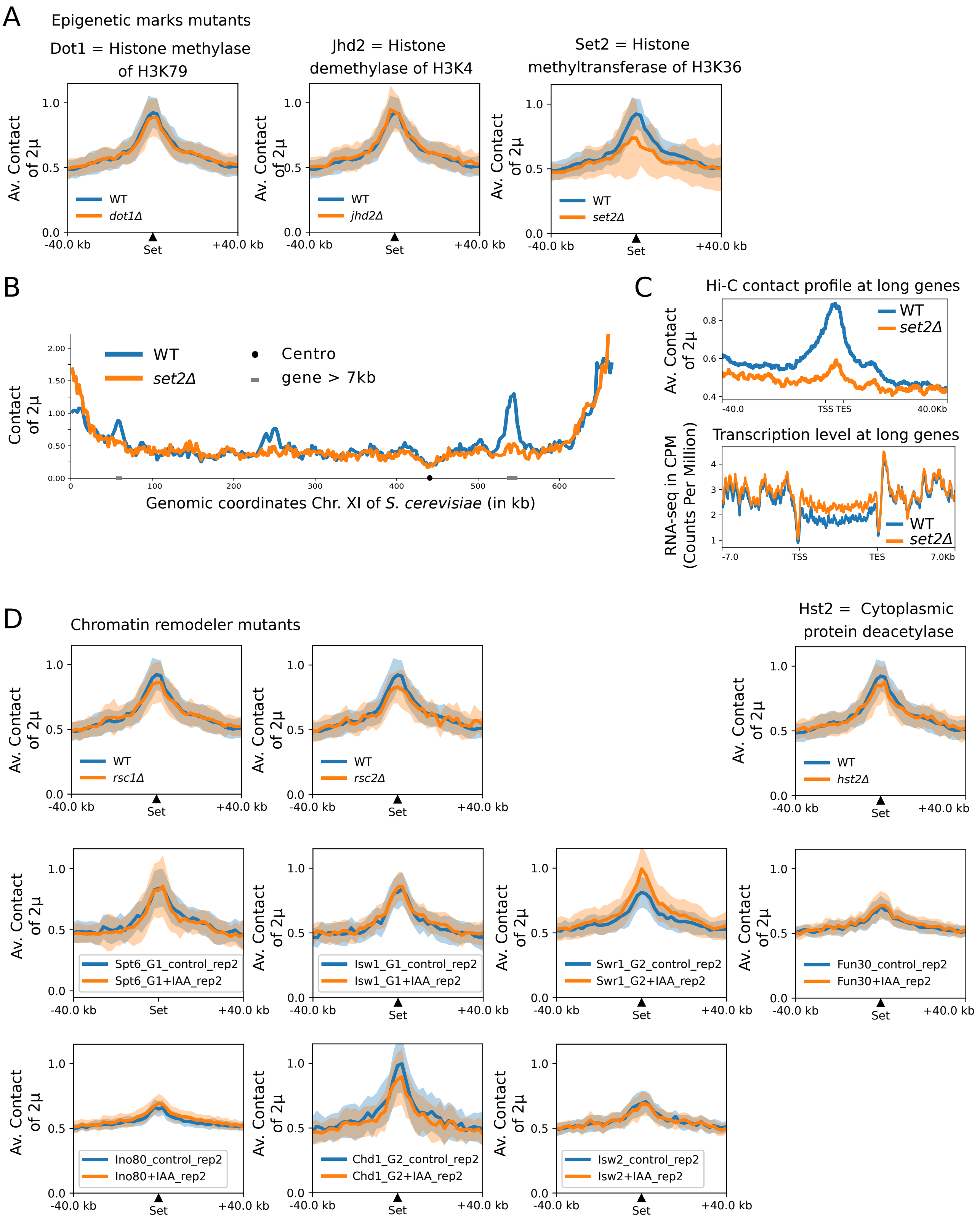

Appendix Figure S12: Contact signal of 2*µ* plasmid in epigenetic marks and chromatin remodelers mutants.

A, Averaged 2*µ* *plasmid* contact signal over the set of identified loci contacted by 2*µ* plasmid in WT, log phase condition for dot1Δ, jhd2Δ and set2Δ mutants (same as Figure 3). **B**, Contact signals of 2*µ* plasmid along chromosomes XI and VIII for WT and set2Δ mutant. **C**, Average contact signal (top) and average transcription level (below) at long genes for WT and set2Δ mutant. **D**, Averaged 2*µ* plasmid contact signal over the set of genomic positions identified in WT, log phase condition for rsc1Δ, rsc2Δ, hts2Δ as well as for

7 chromatin remodelers degradation mutants (AID system) and their corresponding control: Spt6, Isw1, Swr1, Fun30, Ino80, Chd1, Isw2 (data from (Jo *et al*, 2021)). The degron was carried out using the AID system with auxin induction (indole-3-acetic acid, or IAA). Measurements were released in asynchronous population or in G1 and G2 phases when specified (Jo *et al*, 2021).

Name	genotype / specie if not *S. cerevisiae*	background/ parent	Origin
BY4741	MATa [cir+] his3Δ1 leu2Δ0 met15Δ0 ura3Δ0	S288C	Brachmann et al. 1998
BY4742	MATα [cir+] his3Δ1 leu2Δ0 lys2Δ0 ura3Δ0	S288C	Brachmann et al. 1998
BY4743	MATa/α [cir+] his3Δ1/his3Δ1 leu2Δ0/leu2Δ0 met15Δ0/MET15 LYS2/lys2Δ0 ura3Δ0/ura3Δ0	S288C	Brachmann et al. 1998
W303	MATa [cir+] leu2-3,112 trp1-1 can1-100 ura3-1 ade2-1 his3-11,15	K6001	R. Rhotstein
Y9-4	[cir-] strain from Indonesia (used for Ragi fermentation, finger millet)	NA	Peter et al.2018
BHB	[cir+] strain from bakeries in Australia	NA	Peter et al. 2018
yTT7177 HHF2	MATa RAD5+ ura3-1 hht1-hhf1::Nat hht2-hhf2::Hyg trp1-1::pRS 404-HHT2-HHF2	W303	Swygert et al 2021
yTT7175 H4 5toA	MATa [cir-] RAD5+ ura3-1 hht1-hhf1::Nat hht2- hhf2::Hyg trp1-1::pRS 404-HHT2-hhf2-K16A,R17A,H18A,R19A,K20A	W303	Swygert et al 2021
RSGY 712	MATa [cir-] leu2-3,112 trp1-1 can1-100 ura3-1 ade2-1 his3-11,15 *M.mycoides*-linear CEN-ARS-HIS3	W303	Chapard et al. 2023
RSGY 976	MATa [cir+] leu2-3,112 trp1-1 can1-100 ura3-1 ade2-1 his3-11,15 pKAN-STB-P	W303	McQuaid et al.2019
RSGY 1056	MATa [cir+] his3Δ1 leu2Δ0 met15Δ0 ura3Δ0 jhd2::KANMX4	BY4741	This study
RSGY 1055	MATa [cir+] his3Δ1 leu2Δ0 met15Δ0 ura3Δ0 set2::KANMX4	BY4741	This study
RSGY 1058	MATa/α [cir+] his3-11,15/his3Δ1 leu2-3,112/leu2Δ0 LYS2/lys2Δ0 ura3-1/ura3Δ0 trp1-1/TRP1 can1-100/CAN1 ade2-1/ADE2 met14/MET14 *M.mycoides*-linear CEN-ARS-HIS3/	BY4741XRSGY 712	This study
RSGY 1059	MATa [cir-] his3Δ1 leu2Δ0 met15Δ0 ura3Δ0	BY4741	This study
RSGY 1065	Y9-4 [cir-] pKAN-STB-P	Y9-4	This study
RSGY 1068	MATa [cir-] his3Δ1 leu2Δ0 met15Δ0 ura3Δ0 pKAN-ΔREP1	BY4741/ RSGY 1059	This study
RSGY 1069	MATa [cir-] his3Δ1 leu2Δ0 met15Δ0 ura3Δ0 pKAN-ΔSTB-P	BY4741/ RSGY 1059	This study
RSGY	MATa [cir-] his3Δ1 leu2Δ0 met15Δ0 ura3Δ0 pKAN-STB-P	BY4741/	This study

1070		RSGY 1059	
RSGY 1116	MATa [cir+] his3Δ1 leu2Δ0 met15Δ0 ura3Δ0 dot1 ::KANMX4	BY4741	This study
RSGY 1217	MATa [cir+] his3Δ1 leu2Δ0 met15Δ0 ura3Δ0 hst2::KANMX4	BY4741	This study
RSGY 1218	MATa [cir-] RAD5+ ura3-1 hht1-hhf1::Nat hht2- hhf2::Hyg trp1-1::pRS 404-HHT2-hhf2-K16A,R17A,H18A,R19A,K20A pKAN-STB-P	YTT7175 (W303)	This study
RSGY 1226	MATa [cir+] his3Δ1 leu2Δ0 met15Δ0 ura3Δ0 rsc1 ::KANMX4	BY4741	This study
RSGY 1252	MATa [cir+] his3Δ1 leu2Δ0 met15Δ0 ura3Δ0 rsc2::KANMX4	BY4741	This study
RSGY 1255	MATa [cir-] his3Δ1 leu2Δ0 met15Δ0 ura3Δ0 pARS	BY4741/ RSGY 1059	This study
RSGY 1382	*Lachancea fermentati* CBS6772 [cir+]	NA	Gilles Fisher
RSGY 1383	*Lachancea waltii* VRRL4-8285 [cir+]	NA	Gilles Fisher
AX4	*Dictyostelium discoideum* [cir+]	NA	ATCC 201386
yAT4593	MATa [cir+] leu2-3,112 trp1-1 can1-100 ura3-1 ade2-1 his3-11,15 rap1::RAP1-GFP(ADE2)	W303XRSGY 712	This study
yAT4595	MATα [cir+] leu2-3,112 trp1-1 can1-100 ura3-1 ade2-1 his3-11,15 rap1::RAP1-GFP-ADE2) XVI-*M. mycoides*-fusion	W303XRSGY 712	This study
RSGY 1154	MATa his3Δ200 leu2Δ0 lys2Δ0 trp1Δ63 ura3Δ0 met15Δ0 can1::MFA1pr-HIS3 hht1-hhf1::NatMX4 hht2-hhf2::HHTS-HHFS(del 15-18)-URA3	Boeke-EMH-H4-199	Non Essential Histone H3 & H4 Mutant Collection (Yeast) v2 (Dai et al. 2008)
RSGY 1155	MATa his3Δ200 leu2Δ0 lys2Δ0 trp1Δ63 ura3Δ0 met15Δ0 can1::MFA1pr-HIS3 hht1-hhf1::NatMX4 hht2-hhf2::HHTS-HHFS(del-17-20)-URA3	Boeke-EMH-H4-192	Non Essential Histone H3 & H4 Mutant Collection (Yeast) v2 (Dai et al. 2008)

Appendix Table S1: List of strains used in the present study.

Name	Relevant genetic features	Origin
pKan or pKan-STB-P	kanMX4 2µm flp- ΔSTB-P XhoI STB-P *REP1 REP2 RAF*	McQuaid et al.2019
pKan ΔREP1	kanMX4 2µm flp- STB Δ*REP1 REP2 RAF (derived from* pKan)	McQuaid et al.2019
pKan ΔSTB	kanMX4 2µm flp- ΔSTB-P *REP1 REP2 RAF (derived from* pKan)	McQuaid et al.2019
pRS413	CEN4 ARS *HIS3*	Sikorksi et al. 1989
pARS	ΔCEN4 ARS *HIS3*	This study

Appendix Table S2: List of plasmids used in this study.

Library Id	GEO accession number	Description	Figure	% plasmid seq in the lib
FG0092	GSM7873861	Hi-C of the natural yeast BHB	**Fig. EV2A**	**10%**
FG0093	GSM7873860	Hi-C of the natural yeast Y9 with p*KAN* plasmid	**Fig. EV2A**	**0.2%**
FG0089	GSM7873862	Hi-C set2Δ mutant	**Fig. 3D, Appendix Fig. S12**	**4.6%**
FG0090	GSM7873863	Hi-C jhd2Δ mutant	**Fig. 3D, Appendix Fig. S12**	**3.0%**
FG0095	GSM7873864	Hi-C with the additional chromosome Mmyco	**Fig. 2I, Appendix Fig. S10**	**1.44%**
FG074	GSM7873865	Hi-C p*Kan* plasmid	**Fig 3E, Fig 3F, Fig. EV4**	**0.5%**
FG0105	GSM7873866	Hi-C p*Kan*-ΔSTB	**Appendix Fig. S5**	**0.1%**
FG0106	GSM7873867	Hi-C p*Kan*-Δ*REP1*	**Appendix Fig. S5**	**0.05%**
FG0125	GSM7873868	Hi-C before heat shock	**Fig 1C, Fig 1E, Fig 2A, Fig 2B, Fig 2C, Fig 2F, Fig 2G, Fig. EV3, Appendix Fig. S12**	**3.1%**
FG0126	GSM7873869	Hi-C Heat shock 1min	**Fig. EV3**	**3.2%**
FG0127	GSM7873870	Hi-C Heat shock 2min	**Fig. EV3**	**3.2%**
FG0128	GSM7873871	Hi-C Heat shock 5min	**Fig 2G, Fig. EV3**	**3.2%**
FG0141	GSM7873873	Hi-C dot1Δ mutant	**Fig 3D**	**4.2%**
FG0166	GSM7873874	Hi-C hst2Δ mutant	**Fig 3D**	**2.5%**
FG0170	GSM7873875	Hi-C 5toA mutant log phase, with pKan plasmid replicate 1	**Fig 3E, Fig 3F,**	**0.6%**
FG0177	GSM7873876	Hi-C pARS plasmid	**Fig 1D, Fig 1E, Fig 3E, Appendix Fig. S3B**	**0.25%**
FG0129	GSM7873881	ChIP_Rep1_input	**Fig 3G, Appendix Fig. S2**	**3.4%**
FG0130	GSM7873882	ChIP_Rep1_IP	**Fig 3G, Appendix Fig. S2**	**28.5%**
AT638	GSM7873879	Hi-C rsc1Δ mutant	**Fig 3D, Appendix Fig. S12**	**3.2%**
AT639	GSM7873880	Hi-C rsc2Δ mutant	**Fig 3D, Appendix Fig. S12**	**0.8%**

FG0635	GSM8446384	Hi-C of *L. waltii*	**Fig 4B**	**6.9%**
FG0634	GSM8446383	Hi-C of *L. fermentati*	**Fig 4B**	**2.0%**
LM308	GSM8446351	RNA-seq of *L. waltii*	**Fig 4B**	**0.5%**
LM309	GSM8446352	RNA-seq of *L. fermentati*	**Fig 4B**	**0.13%**
FG0137	GSM7873872	Hi-C of *D. discoideum*	**Fig 4C**	**4.5%**
FG146	GSM8738741	Hi-C of H4 tail deletion mutant from amid acids 15 to 17	**Fig. EV4H**	**2.7%**
FG147	GSM8738741	Hi-C of H4 tail deletion mutant from amid acids 17 to 20	**Fig. EV4H**	**3.0%**
PL01	GSM8738743	Hi-C of 5toA mutant with pKan plasmid, log phase, replicate 2	**Fig. EV4B**	**0.3%**
FG0688	GSM8741258	Shotgun sequencing of WT after O/N culture, rep 1	**Fig. EV4F**	**0,17%**
FG0689	GSM8741259	Shotgun sequencing of WT after O/N culture, rep 2	**Fig. EV4F**	**0,19%**
FG0690	GSM8741260	Shotgun sequencing of WT after O/N culture, rep 3	**Fig. EV4F**	**0,19%**
FG0679	GSM8741255	Shotgun sequencing of 5toA after O/N culture, rep 1	**Fig. EV4F**	**0,18%**
FG0680	GSM8741256	Shotgun sequencing of 5toA after O/N culture, rep 2	**Fig. EV4F**	**0,20%**
FG0681	GSM8741257	Shotgun sequencing of 5toA after O/N culture, rep 3	**Fig. EV4F**	**0,19%**

Appendix Table S3: Genomic datasets generated in this study.

**Experiment type and condition**	**Figure**	**Ref**	**Identifier**
Micro-C, WT log and quiescence phases	**Fig 1, Fig EV1, Fig EV2, Fig EV4, Appendix Fig. S2**	(Swygert *et al*, 2019)	SRR7939017 SRR7939018
Hi-C, WT W303, G1 arrest	**Fig EV2**	(Piazza *et al*, 2021)	SRR12284705
MicroC, WT A364, asynchronous	**Fig EV2**	(Costantino *et al*, 2020)	SRR11893084
MicroC, mitotic cell cycle	**Fig 1, Appendix Fig. S6**	(Costantino *et al*, 2020)	SRR11893107 to SRR11893114
Hi-C, WT diploid heterozygous	**Fig EV2**	(Piazza *et al*, 2021)	SRR12284704
Hi-C, WT DSB t2 replicate2	**Fig EV2**	(Piazza *et al*, 2021)	SRR12284704
Hi-C, WT, with DMSO	**Fig EV2**	(Jeppsson *et al*, 2022)	SRR13147965
Hi-C, WT, with HU	**Fig EV2**	(Jeppsson *et al*, 2022)	SRR13147975
Micro-C, Mcd1-AID (rep1) and control	**Fig 1, Fig EV2**	(Costantino *et al*, 2020)	SRR11893086 SRR11893085
Hi-C, Smc2-AID and control	**Fig 1, Fig EV2**	(Guérin *et al*, 2019)	SRR9040342 SRR9040345
Hi-C, Smc5-AID, Smc6-AID and control	**Fig EV2**	(Jeppsson *et al*, 2024)	SRR24991174 SRR24991179
Hi-C, Sir3-AID and control	**Fig EV2**	(Ruault *et al*, 2021)	SRR12108219 SRR12108218
Hi-C, Pds5-degron, Eco1-degron, Wapl-degron	**Fig EV2**	(Dauban *et al*, 2020)	SRR10687277 SRR10687278
Hi-C, cdc45-AID and control (alpha-factor G1)	**Fig EV2**	(Dauban *et al*, 2020)	SRR10687274 SRR8769554

Hi-C, Top2 depleted mutant	**Fig EV2**	(Lazar-Stefanita *et al*, 2017)	GSE90902
Hi-C, mutants Chl4Δ, sgo1Δ, nocodazole treatment	**Fig EV2**	(Paldi *et al*, 2020)	SRR8718857, SRR8718853
MicroC, Rad50 mutant and control	**Fig EV2**	(Forey *et al*, 2021)	SRR11489731 SRR11489732
Hi-C, ΔSgs1 DSB t4, ΔSml1 DSB t4 noco, ΔMec3 DSB t4 noco	**Fig EV2**	(Piazza *et al*, 2021)	SRR12284714 SRR12284727 SRR13736493
Hi-C, Fkh mutant and control	**Fig EV2**	(Eser *et al*, 2017)	SRR5337954 SRR5337951
Hi-C, Clb5-Clb6 mutant and control	**Fig EV2**	(Barton *et al*, 2022)	SRR17873477 SRR17873476
Hi-C, Syn6.5 strain and control	**Fig EV2**	(Zhao *et al*, 2022)	SRR22910279 SRR22910280
Hi-C, meiosis (t=0h,3h,4h, 6h)	**Appendix Fig. S6**	(Muller *et al*, 2018)	SRR7126297 SRR7126293 SRR7126301 SRR7340033
Hi-C, meiosis cell cycle, WT	**Appendix Fig. S6**	(Schalbetter *et al*, 2019)	SRR8689946 to SRR8689952
Hi-C, chromatin remodelers mutants (Spt6, Isw1, Swr1, Fun30, Ino80, Chd1, Isw2 and controls)	**Appendix Fig. S12**	(Jo *et al*, 2021)	GSE158336

Appendix Table S4: Contact datasets analysed in the present study.
The last column indicates either the identifier for the raw reads available on the Short Read Archive server (SRA) (https://www.ncbi.nlm.nih.gov/sra) or on Gene Expression Omnibus server (GEO) (https://www.ncbi.nlm.nih.gov/geo).

Experiment type and condition	Figure	Ref	Identifier
Pol II ChIP-seq	**Fig 3**	(Morselli *et al*, 2015)	SRR1916157<br>SRR1916162
H3 ChIP-seq, in Log Rep1	**Fig 3, Appendix Fig. S1**	(Swygert *et al*, 2021)	SRR13736587<br>SRR13736589
RNA-seq	**Fig 1,2 & Appendix Fig. S1**	(Garcia-Luis *et al*, 2019)<br>(Grosjean *et al*, 2022)	SRR14693235<br>SRR7692240
ATAC-seq	**Fig 1, Appendix Fig. S1**	(Lee *et al*, 2018)	SRR6246290
Cse4 ChIP-seq	**Appendix Fig. S1**	(Au *et al*, 2020)	SRR10765000<br>SRR10764999
Scc1 ChIP-seq	**Appendix Fig. S1**	(Verzijlbergen *et al*, 2014)	SRR1103930<br>SRR1103928
Brn1 ChIP-seq	**Appendix Fig. S1**	(Swygert *et al*, 2019)	SRR7175367<br>SRR7175368
∼1300 ChIP-exo covering ∼800 different proteins and genomic signals	**Fig 3**	(Rossi *et al*, 2021)	GSE147927
H3 chemical cleavage	**Fig 3**	(Chereji *et al*, 2018)	SRR5399542
RNA-seq in *Dictyostelium discoideum* (vegetative stage, rep1)	**Fig 4**	(Wang *et al*, 2021)	SRR10133961
RNA-seq in WT and set2Δ	**Appendix Fig. S12**	(Lee *et al*, 2018)	SRR6246300<br>SRR6246302

Appendix Table S5: Genomic datasets (other than contact data) analyzed in the present study.
The last column indicates either the identifier for the raw reads available on the Short Read Archive server (SRA) (https://www.ncbi.nlm.nih.gov/sra).

chrom1	start1	end1	size (in bp)	heights	genes	size of genes (in bp)
chr11	645556.0	662855.0	17299.0	1.7946576062413295	FLO10, NFT1, YKR104W, VBA5, GEX2	3509, 3656, 920, 1748, 1847
chr11	534672.0	547838.0	13166.0	1.5666363371997594	YSR3, DYN1	1214, 12278
chr10	197213.0	202983.0	5770.0	1.517288490889262	YJL113W, YJL114W	4647, 680
chr2	503600.0	525703.0	22103.0	1.516601475619222	YBR134W, CKS1, MEC1, YBR137W, YBR138C, YBR139W, IRA1	401, 452, 7106, 539, 1574, 1526, 9278
chr12	53936.0	62695.0	8759.0	1.4722292058592616	VPS13	9434
chr3	2898.0	9192.0	6294.0	1.4558195605936413	GEX1, YCL074W	1847, 926
chr2	517050.0	521282.0	4232.0	1.4485680040170101	YBR139W, IRA1	1526, 9278
chr12	1042509.0	1051402.0	8893.0	1.417916686243783	FMP27	7886
chr2	29642.0	34201.0	4559.0	1.4160211858785632	YBL100W-A, YBL100W-B	1316, 5313
chr4	892112.0	906594.0	14482.0	1.3835652769151738	UPC2, AHA1, YDR215C, ADR1, RAD9, SPR28, MFB1	2741, 1052, 413, 3971, 3929, 1271, 1397
chr2	49507.0	58095.0	8588.0	1.3783353034105281	TEL1, AVT5	8363, 1379
chr1	1835.0	19109.0	17274.0	1.3067575577758859	TDA8, YAL064W-B, YAL065C, YAL066W, SEO1, YAL067W-A, PAU8	380, 380, 386, 308, 1781, 227, 362
chr10	471723.0	511310.0	39587.0	1.3021552124951932	YJR026W, YJR027W, YJR028W, YJR029W, YJR030C, GEA1, CPR7, RAV1, PET191, RAD26, HUL4, YJR037W, YJR038C, YJR039W, GEF1, URB2	1322, 5268, 1322, 5268, 2237, 4226, 1181, 4073, 326, 3257, 2678, 383, 362, 3365, 2339, 3524
chr12	301561.0	317260.0	15699.0	1.2626781675383891	SMC4, CSF1, GAA1	4256, 8876, 1844
chr10	486819.0	494820.0	8001.0	1.2413837107828312	GEA1, CPR7, RAV1	4226, 1181, 4073
chr11	6870.0	11314.0	4444.0	1.210690176567508	FRE2, MCH2	2135, 1421
chr12	960266.0	987370.0	27104.0	1.2011097342615364	YLR419W, URA4, RPN13, YLR422W, ATG17, SPP382,	4307, 1094, 470, 5798, 1253, 2126, 3923

					TUS1	
chr7	560751.0	573875.0	13124.0	1.1920359686605242	YGR038C-A, YGR038C-B, ORM1	1322, 5268, 668
chr10	731309.0	742000.0	10691.0	1.1636826997586747	HXT16, SOR1, MPH3	1703, 1073, 1808
chr16	758368.0	767339.0	8971.0	1.139595309481282	RRG8, YPR117W	833, 7469
chr7	678283.0	690466.0	12183.0	1.1260431103052944	ASK10, ESP1, TEL2	3440, 4892, 2066
chr8	530439.0	549519.0	19080.0	1.1175053063369984	YHR212C, YHR212W-A, YHR213W, YHR213W-A, YHR213W-B, YHR214C-B, YHR214C-C, YHR214W, YHR214W-A	335, 203, 596, 233, 299, 5382, 1436, 611, 485
chr10	690194.0	696100.0	5906.0	1.1153336542341297	HIR3, YJR140W-A	4946, 149
chr12	940797.0	947788.0	6991.0	1.1107531435081044	VIP1, YLR410W-A, YLR410W-B, CTR3	3440, 1316, 5313, 725
chr13	675230.0	683922.0	8692.0	1.110747014393658	YMR206W, HFA1	941, 6371
chr15	689948.0	711459.0	21511.0	1.1060224667726668	IES4, SPR1, ULS1, THI72, YOR192C-A, YOR192C-B, YOR192C-C, PEX27	350, 1337, 4859, 1799, 1316, 5313, 236, 1130
chr12	973000.0	982645.0	9645.0	1.0744155664246269	SPP382	2126
chr3	177161.0	185958.0	8797.0	1.0588576696264722	RPS14A, BPH1	720, 6503
chr14	519095.0	523855.0	4760.0	1.0512697019286774	YNL054W-A, YNL054W-B	1322, 5250
chr13	172335.0	204190.0	31855.0	1.0341535800015351	YMD8, YML039W, YML040W, VPS71, CAT2, RRN11, YML045W, YML045W-A, PRP39, PRM6, YML047W-A, GSF2, RSE1, AIM32, GAL80	1328, 5268, 1322, 842, 2012, 1523, 5268, 1322, 1889, 1058, 365, 1211, 4085, 935, 1307
chr8	24951.0	31724.0	6773.0	1.021036296008452	VMR1, MUP3, YHL037C	4778, 1640, 401
chr14	745672.0	759518.0	13846.0	1.0201124220691007	YNR063W, YNR064C, YNR065C,	1823, 872, 3350, 1310, 3353

					YNR066C, DSE4	
chr9	104114.0	112960.0	8846.0	1.0117802035977008	TAO3, ASG1	7130, 2894
chr11	55317.0	62491.0	7174.0	0.9959933802269895	TOR2, EAP1	7424, 1898
chr15	141184.0	155967.0	14783.0	0.990505363965794	DUF1, MPD2, HAL9, MSH2, SPO21, YPQ1, TRM10, RFC4	3350, 833, 3092, 2894, 1829, 926, 881, 971
chr2	748833.0	757139.0	8306.0	0.9846201663331993	CHK1, RIF1	1583, 5750
chr15	172268.0	181736.0	9468.0	0.9755293057664283	YOL079W, REX4, IRA2	398, 869, 9239
chr4	1095661.0	1105741.0	10080.0	0.9739661553442222	YDR316W-A, YDR316W-B, HIM1, MCM21, YFT2	1322, 5268, 1244, 1189, 824
chr15	1074761.0	1084000.0	9239.0	0.9694758602917287	YOR389W, FEX1, HSP33, YOR392W, ERR1, PAU21	1874, 1127, 713, 443, 1313, 494
chr15	1066201.0	1070220.0	4019.0	0.96895571890689	PHR1, YOR387C	1697, 620
chr3	80516.0	87436.0	6920.0	0.968254308492243	YCL020W, YCL021W-A, YCL022C, KCC4	1316, 377, 515, 3113
chr3	268834.0	278473.0	9639.0	0.9663055113904772	FIG2, YCR090C, KIN82, MSH3	4829, 548, 2162, 3056
chr14	173592.0	179812.0	6220.0	0.9656899698476155	MPA43, RAD50	1628, 3938
chr12	994239.0	999025.0	4786.0	0.9643953456908892	SEN1	6695
chr4	974635.0	994978.0	20343.0	0.9434034129909293	YAP6, SWM1, EXG2, YDR261C-C, YDR261C-D, YDR261W-A, YDR261W-B, YDR262W, DIN7	1151, 512, 1688, 1322, 4815, 1316, 5313, 818, 1292
chr13	822472.0	832376.0	9904.0	0.9258188501580311	PRM15, YMR279C, CAT8	1868, 1622, 4301
chr16	229473.0	239606.0	10133.0	0.9235827292182017	MLH3, SET6, ATG29, REV3, YPL168W, MEX67	2147, 1121, 641, 4514, 1292, 1799
chr4	1023000.0	1028505.0	5505.0	0.9170773496208093	YDR282C, GCN2	1244, 4979
chr14	146366.0	162345.0	15979.0	0.9096426476848459	SIP3, DSL1, ATX1, LTO1, ORC5, POL2, YIF1	3689, 2264, 221, 596, 1439, 6668, 944
chr15	968782.0	975255.0	6473.0	0.9096301010627623	YOR343W-A, YOR343W-B	1316, 5313
chr4	656768.0	674723.0	17955.0	0.906506624766734	YDR102C, STE5, SPO71, TMS1, ARP10, TMN2, TRS85,	332, 2753, 3737, 1421, 854, 2018, 2096, 2147

					YDR109C	
chr7	1029337.0	1040851.0	11514.0	0.9045812003174881	YTA7, EFG1, SLH1, YGR273C, TAF1	4139, 701, 5903, 524, 3200
chr4	817430.0	829543.0	12113.0	0.8999679917732832	UBC1, SDH4, CSN9, YDR179W-A, SCC2, SAS4, CDC1, YDR182W-A	647, 545, 488, 1391, 4481, 1445, 1475, 203
chr13	635035.0	645121.0	10086.0	0.8976219653056883	YMR187C, MRPS17, GCV2, SGS1	1295, 713, 3104, 4343
chr4	869403.0	885407.0	16004.0	0.8945349463684018	MSS4, YDR209C, YDR210C-C, YDR210C-D, YDR210W, YDR210W-A, YDR210W-B	2339, 413, 1322, 5268, 227, 1316, 5313
chr10	40808.0	49483.0	8675.0	0.8898974526013246	YJL206C, LAA1	2276, 6044
chr4	22542.0	28930.0	6388.0	0.8802804057077928	ADY3, LRG1	2372, 3053
chr4	754578.0	763254.0	8676.0	0.861177098292259	YDR149C, NUM1	707, 8246
chr2	629837.0	645177.0	15340.0	0.8578885699555288	COS111, LDH1, KTR3, YBR206W, FTH1, DUR1,2, YBR209W	2774, 1127, 1214, 323, 1397, 5507, 317
chr13	612276.0	616433.0	4157.0	0.8566248142813039	ECM5	4235
chr8	300642.0	315428.0	14786.0	0.8530342193705103	TRA1, GEP4, BIG1	11234, 557, 1094
chr4	510072.0	520570.0	10498.0	0.843611489202025	LYS14, YDR034C-A, YDR034C-C, YDR034C-D	2372, 176, 1316, 5313
chr2	434340.0	442830.0	8490.0	0.8397784948399364	RXT2, YBR096W, VPS15, MMS4	1292, 692, 4364, 2075
chr11	24897.0	29146.0	4249.0	0.8374926554203802	OXP1, URA1	3860, 944
chr8	420574.0	438818.0	18244.0	0.836415830598337	YAP1801, MPC2, SOL3, DNA2, PRP8, YHR165W-A, CDC23	1913, 389, 749, 4568, 7241, 311, 1880
chr3	166916.0	172088.0	5172.0	0.8250770984848462	RHB1, FEN2	629, 1538
chr12	760216.0	777671.0	17455.0	0.8242407342645152	MRPL15, SPH1, CDC3, NKP2, TAD3, YLR317W, EST2, BUD6, MMS22, SFH1	761, 1592, 1562, 461, 1092, 434, 2654, 2366, 4364, 1280

chr14	121894.0	129750.0	7856.0	0.8208003678673955	BNI1, SEC2, TOF1	5861, 2279, 3716
chr12	750229.0	754499.0	4270.0	0.8189369528404543	IMH1, CDC25	2735, 4769
chr4	732353.0	738245.0	5892.0	0.8156378152097482	HPR1, RUB1, MTQ2, DOP1	2258, 306, 665, 5096
chr16	431703.0	450167.0	18464.0	0.8092247877389132	PDR12, GRX5, YPL060C-A, MFM1, ALD6, YPL062W	4535, 452, 5409, 1241, 1502, 404
chr15	117859.0	125473.0	7614.0	0.8069931225025249	ITR2, YOL103W-A, YOL103W-B	1829, 1322, 5268
chr3	146208.0	153479.0	7271.0	0.8031658090052879	SRD1, YCR018C-A, MAK32	665, 254, 1091

Appendix Table S6: Set of the 73 hotspots of contact of 2µ plasmid automatically detected in WT, log phase condition.

Set the 73 hot spots of contact detected in WT, log phase condition from MicroC data (Swygert *et al*, 2019). Size and height correspond to the size (in bp) and maximum height of the detected peak of contact of 2µ plasmid. The column genes correspond to the names of the genes present in the detected peak region. The column Size of genes gives the size (in bp) of the genes present in the detected region.

References

Au W-C, Zhang T, Mishra PK, Eisenstatt JR, Walker RL, Ocampo J, Dawson A, Warren J, Costanzo M, Baryshnikova A, *et al* (2020) Skp, Cullin, F-box (SCF)-Met30 and SCF-Cdc4-Mediated Proteolysis of CENP-A Prevents Mislocalization of CENP-A for Chromosomal Stability in Budding Yeast. *PLoS Genet* 16: e1008597

Barton RE, Massari LF, Robertson D & Marston AL (2022) Eco1-dependent cohesin acetylation anchors chromatin loops and cohesion to define functional meiotic chromosome domains. *eLife* 11: e74447

Chereji RV, Ramachandran S, Bryson TD & Henikoff S (2018) Precise genome-wide mapping of single nucleosomes and linkers in vivo. *Genome Biol* 19: 19

Costantino L, Hsieh T-HS, Lamothe R, Darzacq X & Koshland D (2020) Cohesin residency determines chromatin loop patterns. *eLife* 9: e59889

Dai J, Hyland EM, Yuan DS, Huang H, Bader JS & Boeke JD (2008) Probing nucleosome function: a highly versatile library of synthetic histone H3 and H4 mutants. *Cell* 134: 1066–1078

Dauban L, Montagne R, Thierry A, Lazar-Stefanita L, Bastié N, Gadal O, Cournac A, Koszul R & Beckouët F (2020) Regulation of Cohesin-Mediated Chromosome Folding by Eco1 and Other Partners. *Mol Cell*

Eser U, Chandler-Brown D, Ay F, Straight AF, Duan Z, Noble WS & Skotheim JM (2017) Form and function of topologically associating genomic domains in budding yeast. *Proc Natl Acad Sci U S A* 114: E3061–E3070

Forey R, Barthe A, Tittel-Elmer M, Wery M, Barrault M-B, Ducrot C, Seeber A, Krietenstein N, Szachnowski U, Skrzypczak M, *et al* (2021) A Role for the Mre11-Rad50-Xrs2 Complex in Gene Expression and Chromosome Organization. *Mol Cell* 81: 183-197.e6

Garcia-Luis J, Lazar-Stefanita L, Gutierrez-Escribano P, Thierry A, Cournac A, García A, González S, Sánchez M, Jarmuz A, Montoya A, *et al* (2019) FACT mediates cohesin function on chromatin. *Nat Struct Mol Biol* 26: 970–979

Grosjean N, Le Jean M, Armengaud J, Schikora A, Chalot M, Gross EM & Blaudez D (2022) Combined omics approaches reveal distinct responses between light and heavy rare earth elements in Saccharomyces cerevisiae. *J Hazard Mater* 425: 127830

Guérin TM, Béneut C, Barinova N, López V, Lazar-Stefanita L, Deshayes A, Thierry A, Koszul R, Dubrana K & Marcand S (2019) Condensin-Mediated Chromosome Folding and Internal Telomeres Drive Dicentric Severing by Cytokinesis. *Mol Cell* 75: 131-144.e3

Jeppsson K, Pradhan B, Sutani T, Sakata T, Igarashi MU, Berta DG, Kanno T, Nakato R, Shirahige K, Kim E, *et al* (2024) Loop-extruding Smc5/6 organizes transcription-induced positive DNA supercoils. *Mol Cell* 84: 867-882.e5

Jeppsson K, Sakata T, Nakato R, Milanova S, Shirahige K & Björkegren C (2022) Cohesin-dependent chromosome loop extrusion is limited by transcription and stalled replication forks. *Sci Adv* 8: eabn7063

Jo H, Kim T, Chun Y, Jung I & Lee D (2021) A compendium of chromatin contact maps reflecting regulation by chromatin remodelers in budding yeast. *Nat Commun* 12: 6380

Lazar-Stefanita L, Scolari VF, Mercy G, Muller H, Guérin TM, Thierry A, Mozziconacci J & Koszul R (2017) Cohesins and condensins orchestrate the 4D dynamics of yeast chromosomes during the cell cycle. *EMBO J*

Lee S, Oh S, Jeong K, Jo H, Choi Y, Seo HD, Kim M, Choe J, Kwon CS & Lee D (2018) Dot1 regulates nucleosome dynamics by its inherent histone chaperone activity in yeast. *Nat Commun* 9: 240

Matthey-Doret C, Baudry L, Breuer A, Montagne R, Guiglielmoni N, Scolari V, Jean E, Campeas A, Chanut PH, Oriol E, *et al* (2020) Computer vision for pattern detection in chromosome contact maps. *Nat Commun* 11: 5795

McQuaid ME, Polvi EJ & Dobson MJ (2019) DNA sequence elements required for partitioning competence of the Saccharomyces cerevisiae 2-micron plasmid STB locus. *Nucleic Acids Res* 47: 716–728

Morselli M, Pastor WA, Montanini B, Nee K, Ferrari R, Fu K, Bonora G, Rubbi L, Clark AT, Ottonello S, *et al* (2015) In vivo targeting of de novo DNA methylation by histone modifications in yeast and mouse. *eLife* 4: e06205

Muller H, Scolari VF, Agier N, Piazza A, Thierry A, Mercy G, Descorps-Declere S, Lazar-Stefanita L, Espeli O, Llorente B, *et al* (2018) Characterizing meiotic chromosomes' structure and pairing using a designer sequence optimized for Hi-C. *Mol Syst Biol* 14: e8293

Paldi F, Alver B, Robertson D, Schalbetter SA, Kerr A, Kelly DA, Baxter J, Neale MJ & Marston AL (2020) Convergent genes shape budding yeast pericentromeres. *Nature* 582: 119–123

Piazza A, Bordelet H, Dumont A, Thierry A, Savocco J, Girard F & Koszul R (2021) Cohesin regulates homology search during recombinational DNA repair. *Nat Cell Biol* 23: 1176–1186

Rossi MJ, Kuntala PK, Lai WKM, Yamada N, Badjatia N, Mittal C, Kuzu G, Bocklund K, Farrell NP, Blanda TR, *et al* (2021) A high-resolution protein architecture of the budding yeast genome. *Nature* 592: 309–314

Ruault M, Scolari VF, Lazar-Stefanita L, Hocher A, Loïodice I, Koszul R & Taddei A (2021) Sir3 mediates long-range chromosome interactions in budding yeast. *Genome Res*

Sánchez-Gaya V, Casaní-Galdón S, Ugidos M, Kuang Z, Mellor J, Conesa A & Tarazona S (2018) Elucidating the Role of Chromatin State and Transcription Factors on the Regulation of the Yeast Metabolic Cycle: A Multi-Omic Integrative Approach. *Front Genet* 9: 578

Schalbetter SA, Fudenberg G, Baxter J, Pollard KS & Neale MJ (2019) Principles of meiotic chromosome assembly revealed in S. cerevisiae. *Nat Commun* 10: 4795

Swygert SG, Kim S, Wu X, Fu T, Hsieh T-H, Rando OJ, Eisenman RN, Shendure J, McKnight JN & Tsukiyama T (2019) Condensin-Dependent Chromatin Compaction Represses Transcription Globally during Quiescence. *Mol Cell* 73: 533-546.e4

Swygert SG, Lin D, Portillo-Ledesma S, Lin P-Y, Hunt DR, Kao C-F, Schlick T, Noble WS & Tsukiyama T (2021) Local chromatin fiber folding represses transcription and loop extrusion in quiescent cells. *eLife* 10: e72062

Verzijlbergen KF, Nerusheva OO, Kelly D, Kerr A, Clift D, de Lima Alves F, Rappsilber J & Marston AL (2014) Shugoshin biases chromosomes for biorientation through condensin recruitment to the pericentromere. *eLife* 3: e01374

Wang SY, Pollina EA, Wang I-H, Pino LK, Bushnell HL, Takashima K, Fritsche C, Sabin G, Garcia BA, Greer PL, *et al* (2021) Role of epigenetics in unicellular to multicellular transition in Dictyostelium. *Genome Biol* 22: 134

Zhao Y, Coelho C, Hughes AL, Lazar-Stefanita L, Yang S, Brooks AN, Walker RSK, Zhang W, Lauer S, Hernandez C, *et al* (2022) Debugging and consolidating multiple synthetic chromosomes reveals combinatorial genetic interactions. 2022.04.11.486913 doi:10.1101/2022.04.11.486913 [PREPRINT]
