# Supplementary figures and images for: Parasitic plasmids are anchored to inactive regions of eukaryotic chromosomes through a nucleosome signal
Source: EMBO J. 2025 Feb 27;44(7):2134–56. doi: 10.1038/s44318-025-00389-1 (PMC11962162; doi:10.1038/s44318-025-00389-1)

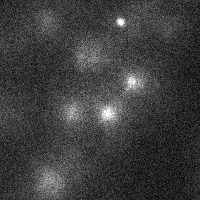

Supplement: Supplementary file 3 — Source data Fig. 2 [file 44318_2025_389_MOESM3_ESM.zip › SD figure 2I/Figure-2I_Fusion_XVI_mycoides_Zstacks_3colors.tif]

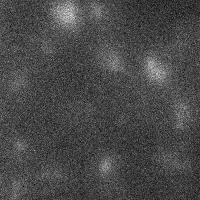

Supplement: Supplementary file 3 — Source data Fig. 2 [file 44318_2025_389_MOESM3_ESM.zip › SD figure 2I/Figure-2I_WT_Zstacks_3colors.tif]
