# Supplementary material for: Parasitic plasmids are anchored to inactive regions of eukaryotic chromosomes through a nucleosome signal
Source: EMBO J. 2025 Feb 27;44(7):2134–56. doi: 10.1038/s44318-025-00389-1 (PMC11962162; doi:10.1038/s44318-025-00389-1)
Supplement: Supplementary file 6 — Expanded View Figures [file 44318_2025_389_MOESM6_ESM.pdf]

Expanded View Figures

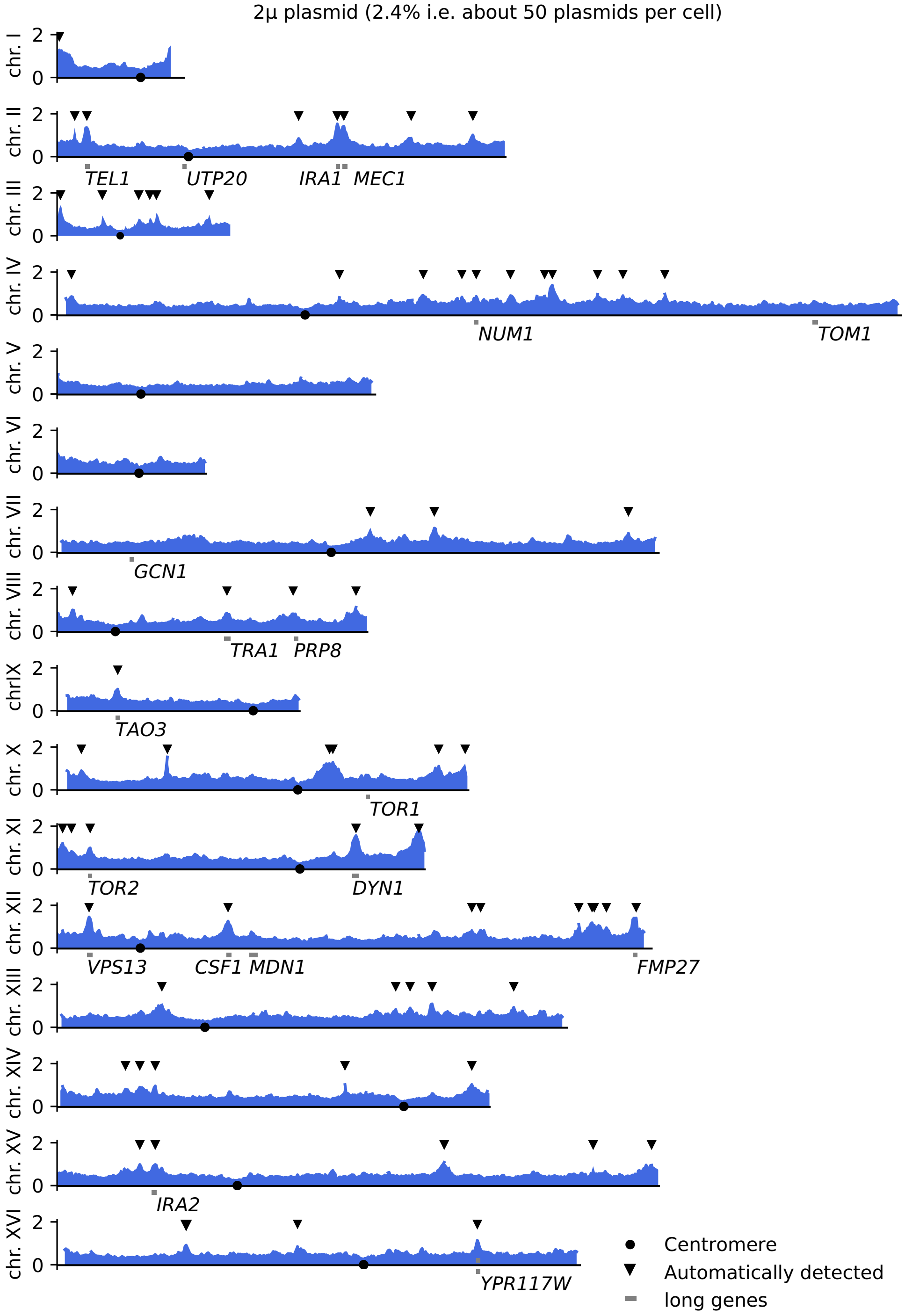

Figure EV1. Contact signal of the 2μ plasmid along the 16 chromosomes of *S. cerevisiae*.

The contact signal is binned at 2 kb, genes with size >7 kb are annotated with grey rectangles and their names (MicroC data from Swygert et al (2019)). Automatically detected peaks of contact (73 genomic positions) were annotated with black triangles.

© The Author(s)

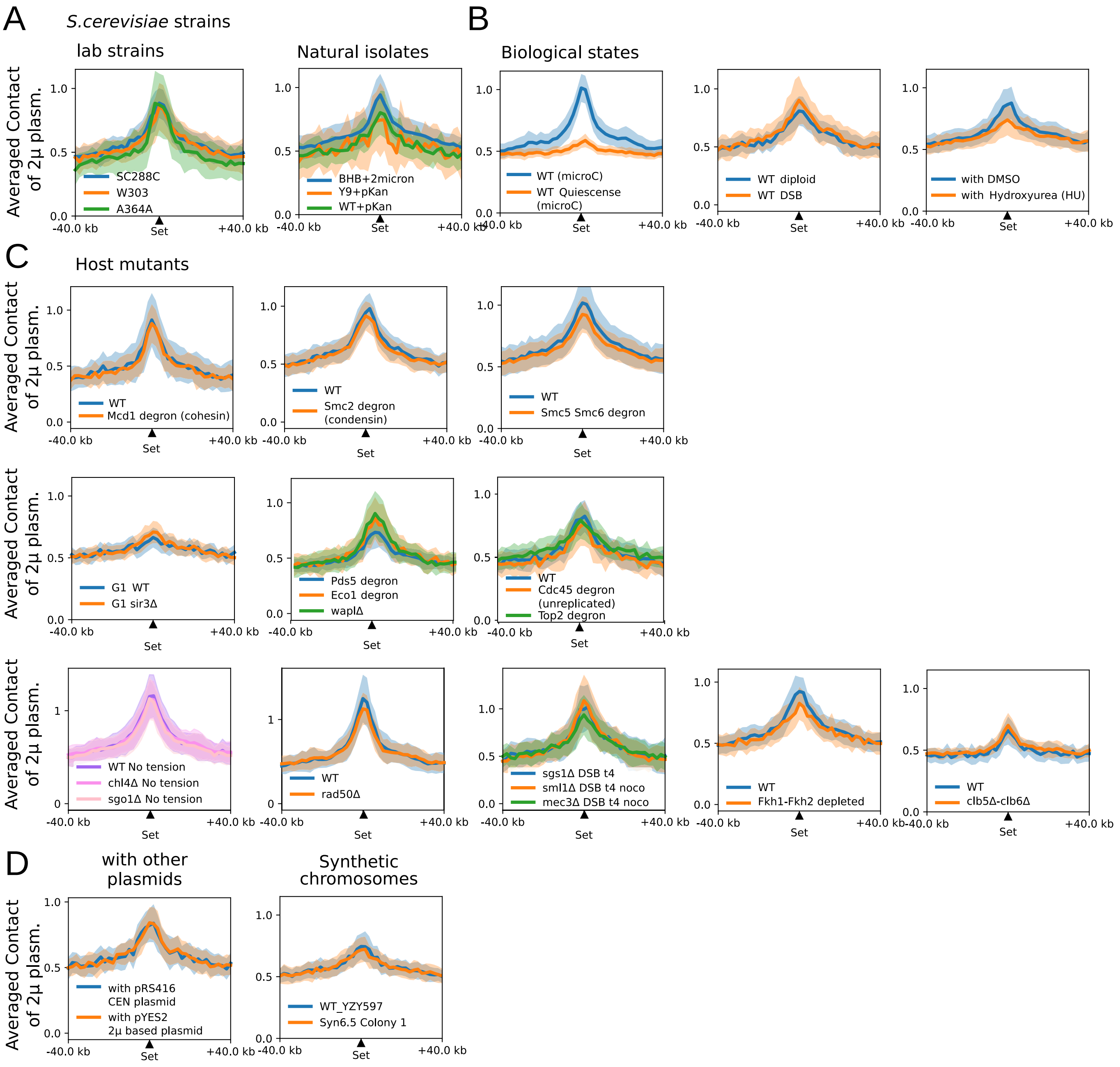

Figure EV2. The specific positioning of the 2µ plasmid is conserved under a wide variety of biological conditions and mutants.

(**A**) Averaged 2µ plasmid contact signal over the hotspots of contact identified in WT, log phase in different lab strains of *S. cerevisiae*: SC288C, W303 (Dauban et al 2020), A364A (Costantino et al, 2020) and strains from natural isolates (Peter et al, 2018). pKan version of the plasmid was used for the Y9 strain to ensure stability as well the corresponding control. pKan contains the KAN resistance gene and *FLP1* gene is disrupted. (**B**) Averaged 2µ plasmid contact signal over the hotspots of contact identified in WT, log phase in different biological states: in quiescence (Swygert et al, 2019) (same as Fig. 1C), in diploid stage, with double-strand break (DSB) of DNA (Piazza et al, 2021), with DMSO or HU treatment (Jeppsson et al, 2022). (**C**) Averaged 2µ plasmid contact signal over the hotspots of contact identified in WT, log phase in different mutants of *S. cerevisiae*: Mcd1 degron mutant (subunit of cohesin, AID system) (Costantino et al, 2020), Smc2 degron mutant (subunit of condensin, AID system) (Guérin et al, 2019), Smc5-Smc6 degron mutant (AID system) (Jeppsson et al, 2024). sir3Δ (Ruault et al, 2021), Pds5, EcoI degron mutants (AID system), wpl1Δ mutant, Cdc45 degron mutant (stopped replication, AID system) (Dauban et al, 2020), Top2 degron mutant (topoisomerase II, AID system) (Lazar-Stefanita et al, 2017), in condition with no tension of microtubules i.e. with nocodazole treatment (noco), chl4Δ, sgo1Δ (Paldi et al, 2020), rad50Δ (Forey et al, 2021). sgs1Δ, sml1Δ, mec3Δ mutants at 4 h after induction of HO endonuclease-mediated site-specific DSB (Piazza et al, 2021), in Fkh1-Fkh2 depleted mutant (GAL1pr-FKH1 fkh2Δ mutant) (Eser et al, 2017), in clb5Δ-clb6Δ mutant (Barton et al, 2022). (**D**) Averaged 2µ plasmid contact signal over the hotspots of contact identified in WT, log phase in presence of other plasmids (centromeric and 2µ based) and with synthetic chromosomes (Zhao et al, 2021).

 © The Author(s)

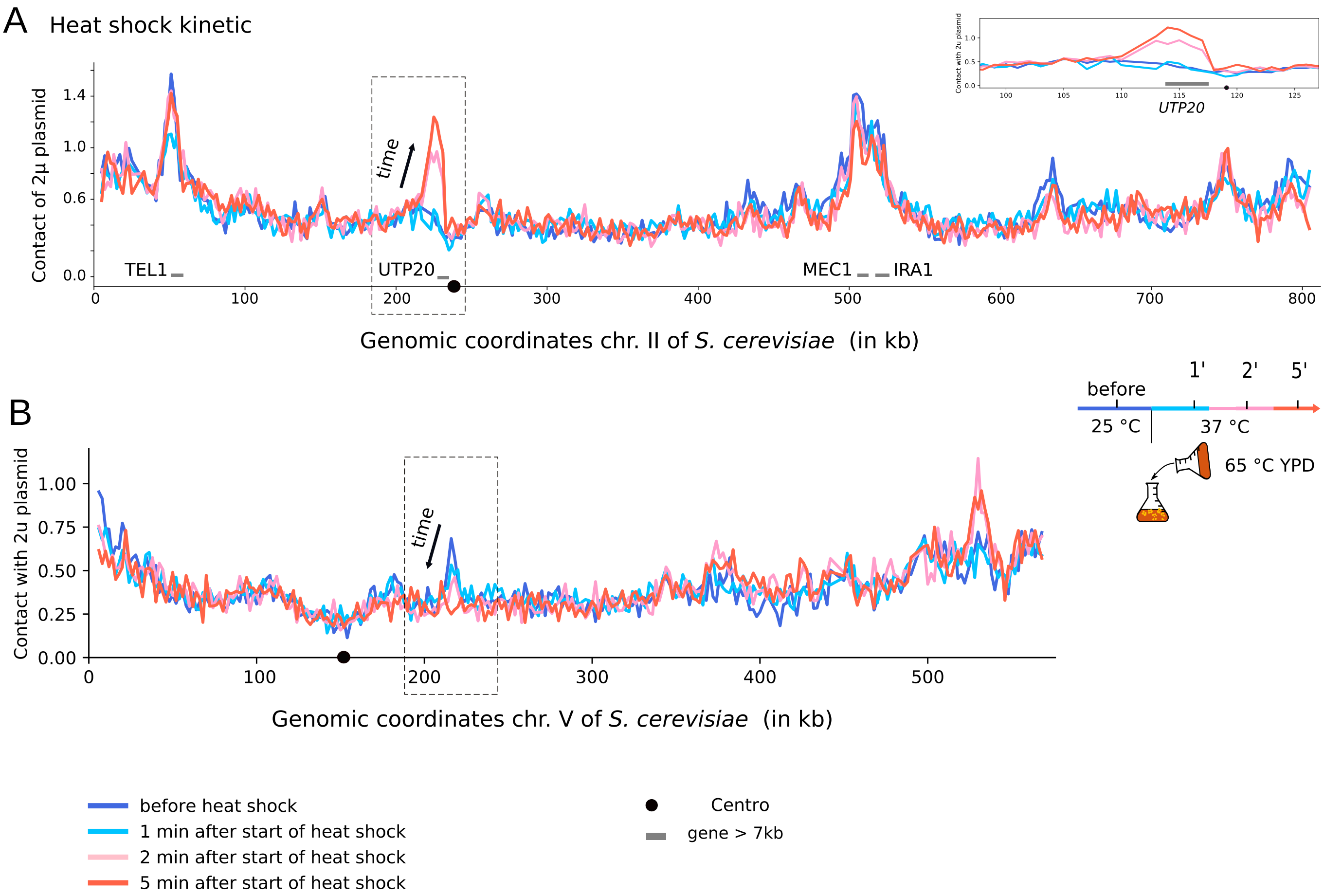

Figure EV3. Contact signal of 2 µ plasmid during a heat shock.

(**A**) Contact signal of 2 µ plasmid along the chromosome II of *S. cerevisiae* for 4 time points: before heat shock, 1 min, 2 min and 5 min after heat shock. (**B**) Contact signal of 2 µ plasmid along the chromosome V of *S. cerevisiae* for 4 time points: before heat shock, 1 min, 2 min and 5 min after heat shock. Binning for contact signals is 2 kb.

© The Author(s)

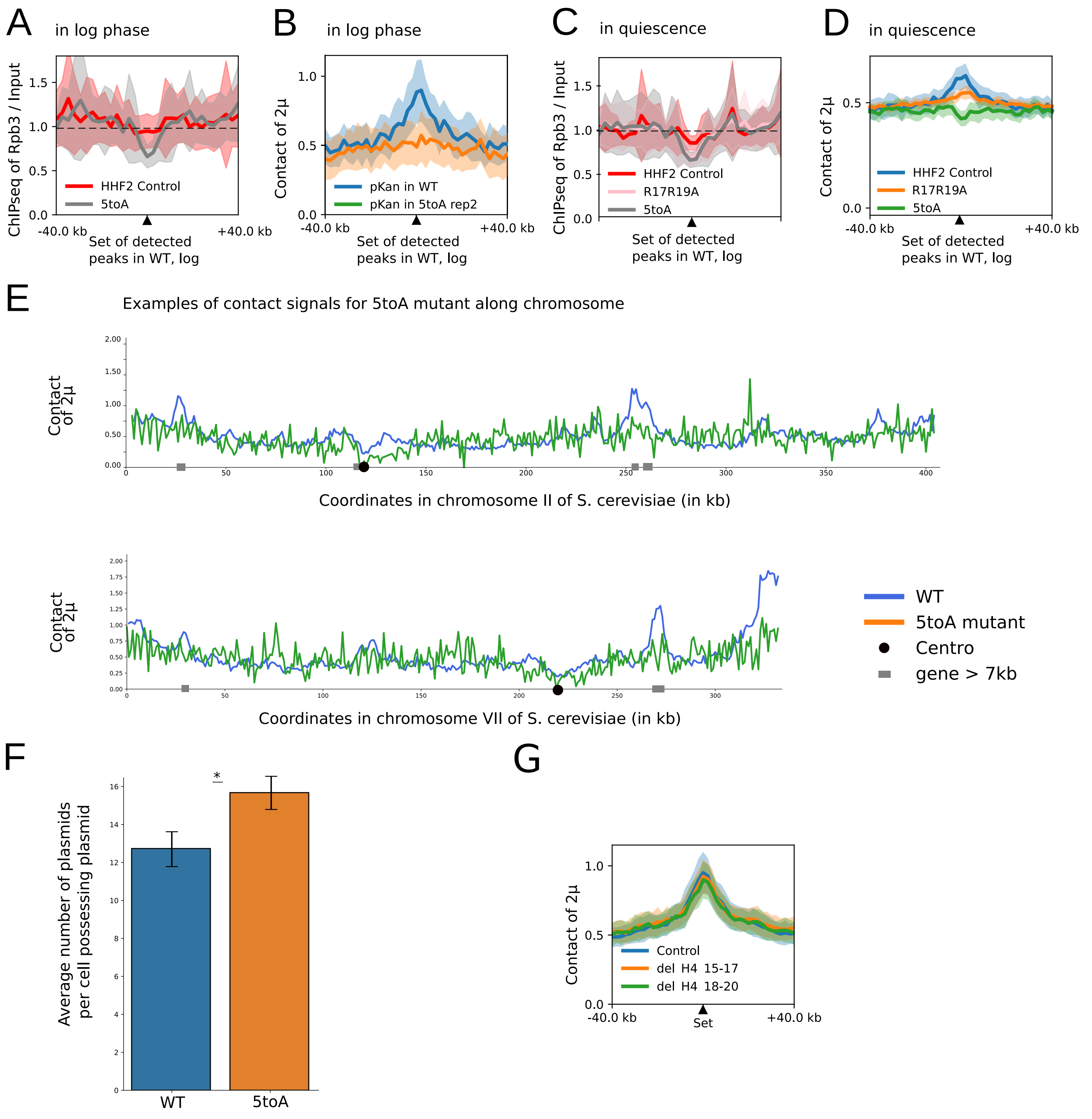
A
in log phase
ChIPseq of Rpb3 / Input
HHF2 Control
5toA
-40.0 kb
+40.0 kb
Set of detected peaks in WT, log
B
in log phase
Contact of 2µ
pKan in WT
pKan in 5toA rep2
-40.0 kb
+40.0 kb
Set of detected peaks in WT, log
C
in quiescence
ChIPseq of Rpb3 / Input
HHF2 Control
R17R19A
5toA
Set of detected peaks in WT, log
D
in quiescence
Contact of 2µ
HHF2 Control
R17R19A
5toA
-40.0 kb
+40.0 kb
Set of detected peaks in WT, log
E
Examples of contact signals for 5toA mutant along chromosome
Contact of 2µ
Coordinates in chromosome II of S. cerevisiae (in kb)
Contact of 2µ
Coordinates in chromosome VII of S. cerevisiae (in kb)
WT
5toA mutant
Centro
gene > 7kb
F
Average number of plasmids per cell possessing plasmid
*
WT
5toA
G
Contact of 2µ
Control
del H4 15-17
del H4 18-20
-40.0 kb
Set
+40.0 kb

 © The Author(s)

Figure EV4. Contact signal of 2µ plasmid in the H4 5toA mutant.

(**A**) Averaged transcription signal measured by Rpb3 (Pol II subunit) ChIP-seq (Swygert et al, 2021) over the set of identified loci contacted by 2µ plasmid in WT, log phase condition for the HHF2 control and H4 5toA mutant in log phase. In the HHF2 control, the endogenous H3 and H4 loci were deleted and complemented by a wild-type copy of H3 and H4 genes at an ectopic locus (TRP1). (**B**) Averaged 2µ plasmid contact signal over the set of identified loci contacted by 2µ plasmid in WT, log phase condition for the WT and H4 5toA mutant in log phase, replicate 2. pKan version of the 2µ plasmid was used to ensure plasmid stability (which contains the *KAN* resistance gene and whose *FLP1* gene is inactivated). (**C**) Averaged transcription signal measured by Rpb3 (Pol II sub-unit) ChIP-seq (Swygert et al, 2021) over the set of identified loci contacted by 2µ plasmid in WT, log phase condition for the HHF2 control, H4 R17R19A and H4 5toA mutants in quiescence phase (Swygert et al, 2021). (**D**) Averaged 2µ plasmid contact signal over the set of identified loci contacted by 2µ plasmid in WT, log phase condition for the HHF2 control, H4 R17R19A and H4 5toA mutants in quiescence phase (Swygert et al, 2021). (**E**) Examples of contact signals of 2µ plasmid (pKan version) in WT and H4 5toA mutant. (**F**) Average number of plasmids per cell computed taking into the proportion of cells having plasmids and reads proportion from plasmid sequence with shotgun sequencing. Results represent the average (± s.d.) from assaying 3 transformants for each condition, respectively, and correspond to overnight culture shown in Fig. 3I (p-value = 0.0308). (**G**), Averaged 2µ plasmid contact signal over the set of identified loci contacted by 2µ plasmid in WT, log phase condition for the control, H4 mutant where the amid acids 15 to 17 have been deleted (del H4 15–17) and H4 mutant where the amid acids 18 to 20 have been deleted (del H4 18–20). Source data are available online for this figure.

© The Author(s)

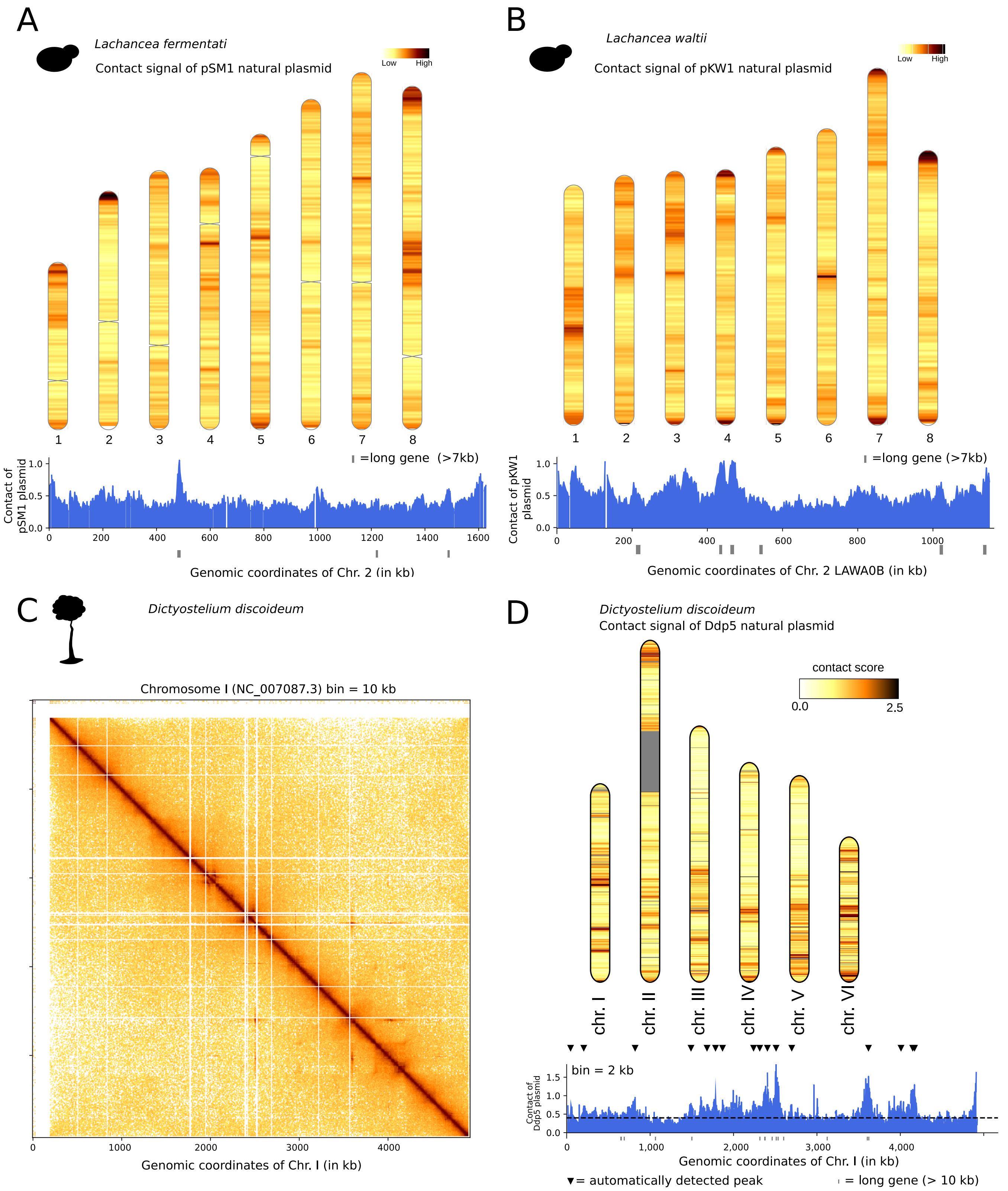
A
Lachancea fermentati
Contact signal of pSM1 natural plasmid
Low High
1 2 3 4 5 6 7 8
=long gene (>7kb)
Contact of pSM1 plasmid
Genomic coordinates of Chr. 2 (in kb)
B
Lachancea waltii
Contact signal of pKW1 natural plasmid
Low High
=long gene (>7kb)
Contact of pKW1 plasmid
Genomic coordinates of Chr. 2 LAWA0B (in kb)
C
Dictyostelium discoideum
Chromosome I (NC_007087.3) bin = 10 kb
Genomic coordinates of Chr. I (in kb)
D
Dictyostelium discoideum
Contact signal of Ddp5 natural plasmid
contact score
0.0 2.5
chr. I chr. II chr. III chr. IV chr. V chr. VI
bin = 2 kb
Contact of Ddp5 plasmid
Genomic coordinates of Chr. I (in kb)
▼= automatically detected peak
= long gene (> 10 kb)

 © The Author(s)

Figure EV5. Contact signals of natural plasmids in other eukaryotes.

(**A**) Contact signal of the pSM1 natural plasmid with the chromosomes of *Lachancea fermentati* yeast. Contact profile for the chromosome 2, long genes are annotated as grey boxes. (**B**) Contact signal of the pKW1 natural plasmid with the chromosomes of *Lachancea waltii* yeast. Contact profile for the chromosome 2, long genes are annotated as grey boxes. (**C**) Contact map of chromosome I of *Dictyostelium discoideum* at 10 kb resolution. (**D**) Contact signal of the Ddp5 natural plasmid with the chromosomes of *Dictyostelium discoideum* and contact profile for chromosome 1. Black triangles indicate automatically detected peaks, and long genes are annotated as grey boxes.

© The Author(s)
